# A Global Assessment of the Chemical Recalcitrance of Seagrass Tissues: Implications for Long-Term Carbon Sequestration

**DOI:** 10.3389/fpls.2017.00925

**Published:** 2017-06-13

**Authors:** Stacey M. Trevathan-Tackett, Peter I. Macreadie, Jonathan Sanderman, Jeff Baldock, Johanna M. Howes, Peter J. Ralph

**Affiliations:** ^1^Climate Change Cluster, University of Technology SydneyUltimo, NSW, Australia; ^2^Centre for Integrative Ecology, School of Life and Environmental Sciences, Deakin UniversityBurwood, VIC, Australia; ^3^Commonwealth Scientific and Industrial Research Organisation Agriculture FlagshipGlen Osmond, SA, Australia; ^4^Woods Hole Research CenterFalmouth, MA, United States

**Keywords:** biogeochemistry, carbon sequestration, global survey, lignocellulose, morphotype, seagrass, recalcitrance, lability

## Abstract

Seagrass ecosystems have recently been identified for their role in climate change mitigation due to their globally-significant carbon sinks; yet, the capacity of seagrasses to sequester carbon has been shown to vary greatly among seagrass ecosystems. The recalcitrant nature of seagrass tissues, or the resistance to degradation back into carbon dioxide, is one aspect thought to influence sediment carbon stocks. In this study, a global survey investigated how the macromolecular chemistry of seagrass leaves, sheaths/stems, rhizomes and roots varied across 23 species from 16 countries. The goal was to understand how this seagrass chemistry might influence the capacity of seagrasses to contribute to sediment carbon stocks. Three non-destructive analytical chemical analyses were used to investigate seagrass chemistry: thermogravimetric analysis (TGA) and solid state ^13^C-NMR and infrared spectroscopy. A strong latitudinal influence on carbon quality was found, whereby temperate seagrasses contained 5% relatively more labile carbon, and tropical seagrasses contained 3% relatively more refractory carbon. Sheath/stem tissues significantly varied across taxa, with larger morphologies typically containing more refractory carbon than smaller morphologies. Rhizomes were characterized by a higher proportion of labile carbon (16% of total organic matter compared to 8–10% in other tissues); however, high rhizome biomass production and slower remineralization in anoxic sediments will likely enhance these below-ground tissues' contributions to long-term carbon stocks. Our study provides a standardized and global dataset on seagrass carbon quality across tissue types, taxa and geography that can be incorporated in carbon sequestration and storage models as well as ecosystem valuation and management strategies.

## Introduction

Seagrasses are considered a biological or ecological group of marine vascular flowering plants that have evolved from three to four separate lineages over the past 70–100 million years (Les et al., 1997). As seagrasses are the essential foundation for coastal ecosystem along most continents, they present as an excellent example of convergent evolution (Les et al., 1997). Although seagrasses have a relatively low diversity (~60 species within 12 genera), they do span a range of sizes, morphologies and life-cycle characteristics (ephemeral vs. persistent, or *r*- and *K*-strategies) (Carruthers et al., 2007). These difference are important for colonization succession of bare sediments (Rasheed, 2004) and in some cases, the particle trapping and accumulation of sediment carbon (Serrano et al., 2016).

Seagrass ecosystems have been identified as carbon (C) sequestration hotspots and thus a critical component in global greenhouse gas mitigation (Laffoley and Grimsditch, 2009). Recent C sequestration research in seagrass ecosystems, i.e., blue carbon research, has aimed to understand what variables influence the ability of seagrass to sequester C permanently within their sediments. Several factors that have been hypothesized to influence this among-meadow variation in C accumulation and preservation, including physical and hydrological conditions (Lavery et al., 2013; Serrano et al., 2016), temperature (Godshalk and Wetzel, 1978), oxygen environment (Mateo et al., 2006), microbial function and metabolism (Duarte et al., 2005), and C source (Mateo et al., 2006; Lavery et al., 2013). However, despite litter quality being a main factor controlling global C remineralization and cycling in terrestrial ecosystems (Couteaux et al., 1995; Silver and Miya, 2001), The influence of litter quality in C sequestration remains comparably understudied for seagrass blue carbon ecosystems (Cebrián et al., 1997; Cebrián, 1999).

Seagrass litter is generally more nutritious to decomposers than terrestrial substrates, but they are relatively nutrient deplete (nitrogen, phosphorus) compared to other sources of marine organic matter and thus can decompose more slowly (Enríquez et al., 1993). Seagrass litter is also more refractory, or resistant to microbial attack, than other marine macrophytes like macroalgae because they retained some of their cell wall structural carbohydrates and lignin from their terrestrial origin (i.e., fiber or lignocellulose ≈ hemicellulose + cellulose + lignin) (Klap et al., 2000), and represent the only source of lignocellulose formed in submerged marine ecosystems (Lewis and Yamamoto, 1990).

The techniques and the taxa and tissues previously used to quantify refractory C in seagrass tissues, however, have been highly variable, and at times inconsistent across studies (reviewed in Table S1), making predictions of seagrass C contributions to sediments unfeasible. For example, much of the quantitative analyses of lignocellulose content in seagrasses have focused on leaf fiber content (hemicellulose + cellulose + lignin) for nutritional herbivory studies (Table S1). As a result, there are sparse data on the structural carbohydrate content of the more lignified below-ground tissues and sheaths (<10 out of 43 studies; Table 1, Table S1). Furthermore, a summary of the available structural carbohydrate data suggest that lignin content is higher in above-ground than below-ground tissues, which is contradictory to histological observations of large seagrass species (e.g., *Posidonia, Thalassodendron*; Table 1) (Kuo and Cambridge, 1978; Cambridge and Kuo, 1982; Barnabas, 1991; Kuo and den Hartog, 2006).

**Table 1:** Summary of structural carbohydrate and fiber content reported from the literature for seagrass tissue types.

**Tissue**	**Structural carbohydrates**			**Fiber**	
	**Cellulose**	**Hemicellulose**	**Lignin**	**NDF**	**ADF**
Leaf (All) *(n = 14 – 43)*	16.84 ± 1.29	12.70 ± 1.24	11.05 ± 1.46	37.59 ± 2.44	26.24 ± 1.62
Leaf Temperate *(n = 1 – 16)*	20.93 ± 3.95	17.07 ± 4.36	12.20 ± 4.82	49.65 ± 1.49	35.10 ± 0
Leaf Tropical *(n = 13 – 27)*	15.75 ± 1.20	11.76 ± 1.14	10.81 ± 1.52	30.44 ± 3.05	25.56 ± 1.58
Non-photosynthetic Above-ground *(n = 1 – 4)*	30.82 ± 7.18	21.00 ± 0	8.80 ± 6.15	N/A	N/A
Rhizome (All) *(n = 4 – 8)*	17.77 ± 1.36	11.75 ± 4.55	3.03 ± 0.78	13.48 ± 3.61	17.83 ± 2.03
Rhizome Temperate *(n = 1 – 2)*	18.30 ± 0	28.90 ± 0	4.45 ± 0.95	N/A	N/A
Rhizome Tropical *(n = 4 – 8)*	17.68 ± 1.61	8.32 ± 3.66	2.56 ± 0.94	13.48 ± 3.61	17.83 ± 2.03
Root (All) *(n = 2 – 4)*	19.34 ± 3.12	34.80 ± 6.10	4.99 ± 0.99	3.09 ± 0.20	N/A
Root Temperate *(n = 1 – 2)*	21.30 ± 0	40.90 ± 0	6.10 ± 0.40	N/A	N/A
Root Tropical *(n = 1 – 4)*	18.37 ± 5.13	28.70 ± 0	3.88 ± 1.82	3.09 ± 0.20	N/A
Rhizome + Root *(n = 11 – 16)*	17.13 ± 2.14	8.02 ± 0.79	11.33 ± 1.19	38.31 ± 2.10	28.02 ± 1.58

*All averages are reported on a % dry weight basis. NDF, neutral detergent fiber; ADF, acid detergent fiber. Full list of species and tissues are in Table S1. A full list of data sources used in the study are provided in the Data Sources section*.

Furthermore, the common wet chemistry techniques, such as proximate analyses to obtain neutral and acid detergent fiber (NDF, ADF), have limitations that may account for these disparate trends in fiber content (Preston et al., 1997; Brinkmann et al., 2002; Hatfield and Fukushima, 2005). ADF can result in impure separation (lignin-bound proteins; Brinkmann et al., 2002) or treatment of ADF with strong acid to quantify lignin (acid detergent lignin) may remove a significant proportion of the desired compound (i.e., terrestrial grasses can lose up to 50% of lignin in acid detergent solution). Alternatively, there are a few techniques for determining structural carbohydrates and lignin content that do not rely on the modification of the cell wall matrix and require minimal preparation, i.e., drying and grinding: Solid state ^13^C- Cross-Polarisation Magic-Angle Spinning (CPMAS) nuclear magnetic resonance (NMR) spectroscopy and diffuse reflectance Fourier-transform infrared (FTIR) spectroscopy (pros and cons reviewed in Hatfield and Fukushima, 2005). Another technique that has been recently used to quantify cell wall components is thermogravimetric analysis (TGA), which differentiates cell wall components based on the temperature at which they are pyrolysized, whereby the loss of mass at higher temperatures is associated with refractory components like lignocellulose content is most useful for the distinction and quantification of labile and refractory components (Capel et al., 2006; Carrier et al., 2011; Pasangulapati et al., 2012). TGA has been used for quantifying labile and refractory C content across blue carbon phyla, including a small subset of Australian seagrass species (Trevathan-Tackett et al., 2015).

To build on this previous work, we surveyed the organic composition of seagrasses via opportunistic seagrass collection, using each of these three non-invasive techniques to provide a multi-proxy approach to quantifying seagrass C quality and structural complexity. We hypothesize that some seagrass samples, such as below-ground tissues or seagrasses with a larger morphological structure, will have higher proportions of refractory C. In collecting seagrasses from across the globe and creating a standardized and comprehensive dataset, our goal is to resolve the variation in seagrass C quality across tissue types, taxa and geography. We expect that these data can be incorporated in models and management strategies that take into account seagrass litter as a key driver that can explain some of the global variability in blue carbon sequestration.

## Methods

### Sample collection and preparation

**Figure 1:**
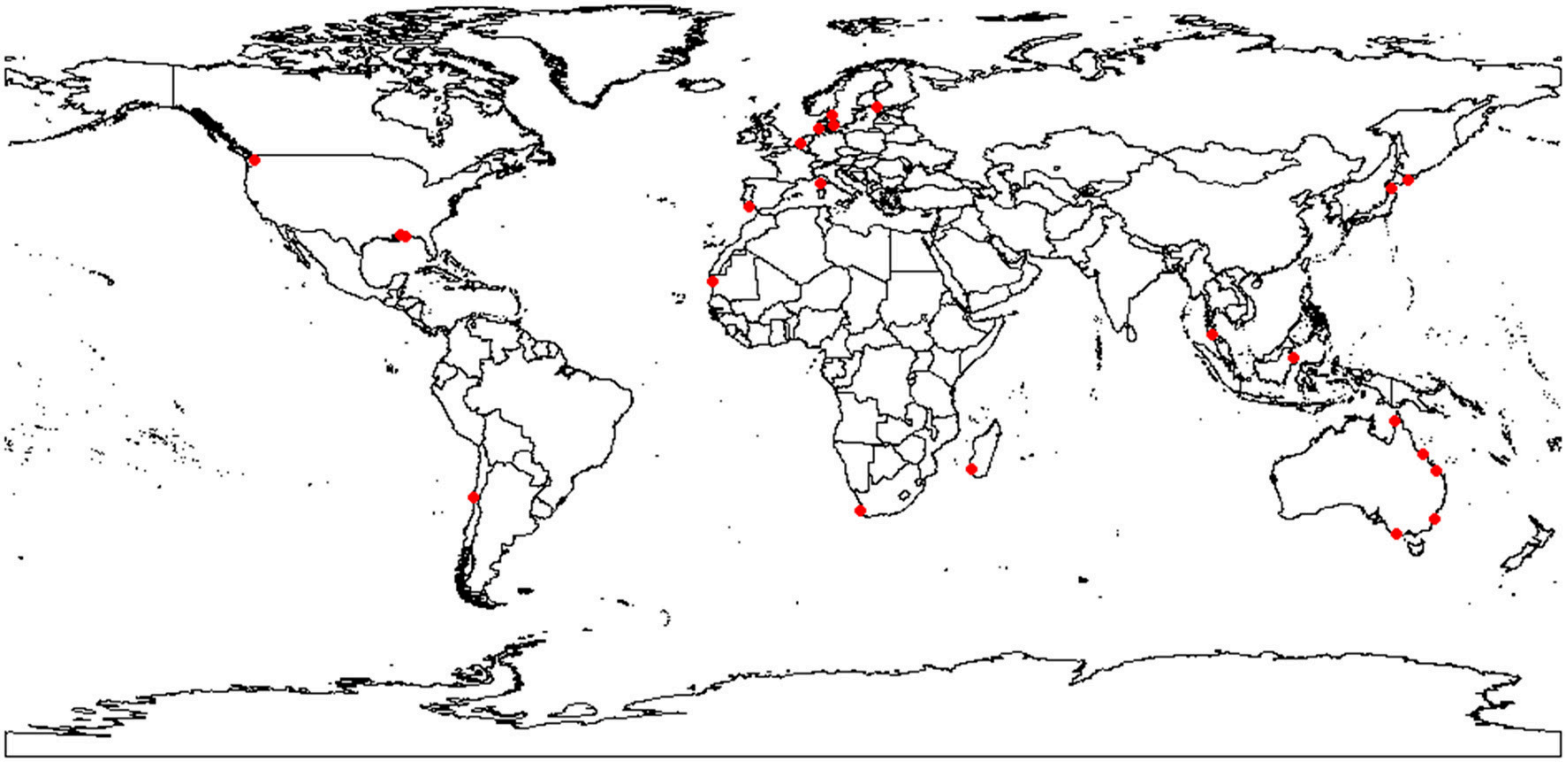
Map of seagrasses collected.

Between August 2012 and March 2015, 23 seagrass species were collaboratively and opportunistically collected from 16 countries (Table 2; Figure 1). Collections occurred across all seasons. After being cleaned of epiphytes and attached infauna and sediments, samples were heat-dried until constant mass and sent to the University of Technology Sydney (UTS) for analysis. Tissue types were separated before being ground to a fine powder using a ball mill (Pulverisette 7, Fritsch, Germany) and stored in a desiccator to avoid moisture reabsorption.

**Table 2:** Summary of the seagrass samples collected and the variables explored: tissue type, taxa and climatic region.

**Family**	**Species**	**Bioregion**	**Country**	**Leaf**	**Rhizome**	**Root**	**Non-photosynthetic above-ground**		
							**Sheath**	**Stem**	**Vertical rhizome**
Cymodoceaceae	*Amphibolis antarctica*	6	Australia^*^^†^	X					
	*Cymodocea nodosa*	1	Portugal^*^^†^	X	X	X			
		2	Mauritania	X	X	X	X		
	*Cymodocea rotundata*	5	Indonesia	X	X	X	X		
			Madagascar^*^	X	X	X			
			Thailand	X	X	X	X		
	*Cymodocea serrulata*	5	Madagascar^*^	X	X	X			
	*Halodule uninervis*	5	Australia^*^^†^	X	X	X			
			Indonesia	X	X	X	X		
			Madagascar	X	X	X			
	*Halodule wrightii*	2	Mauritania	X	X	X			
			USA	X	X	X			
	*Syringodium isoetifolium*	5	Indonesia	X	X	X			
			Madagascar^*^^†^	X	X	X			
	*Thalassodendron ciliatum*	5	Madagascar^*^^†^	X	X	X			X
Hydrocharitaceae	*Enhalus acoroides*	5	Australia^*^^†^	X	X	X	X		
			Thailand^*^	X			X		
	*Halophila australis*	6	Australia	X	X			X	
	*Halophila ovalis*	5	Australia^*^^†^	X	X				
			Indonesia	X	X	X			
			Madagascar	X	X	X			
			Thailand	X	X				
	*Halophila spinulosa*	5	Australia	X	X	X		X	
	*Thalassia hemprichii*	5	Indonesia	X	X	X	X		
			Madagascar^*^^†^	X	X	X			
			Thailand^*^	X	X	X	X		
	*Thalassia testudinum*	2	USA	X	X	X	X		
Posidoniaceae	*Posidonia australis*	6	Australia^*^^†^	X	X	X	X		
	*Posidonia oceanica*	3	Corsica^*^^†^	X	X	X	X		
Ruppiaceae	*Ruppia maritima*	1	Sweden^*^^†^	X	X	X	X		
Zosteraceae	*Phyllospadix iwatensis*	4	Japan	X	X	X	X		
	*Zostera capensis*	6	South Africa	X	X	X			
	*Zostera chilensis*	6	Chile^*^	X	X	X		X	
	*Zostera marina*	1	Sweden^*^	X	X	X	X		
			Denmark	X	X	X	X		
			Finland	X	X	X	X		
			Portugal	X	X	X			
			Germany	X	X	X	X		
		4	Japan^*^	X	X	X	X		
			USA	X	X	X			
	*Zostera muelleri*	5	Australia^†^	X	X	X			
		6	Australia^*^^†^	XX					
	*Zostera nigricaulis*	6	Australia^*^^†^	X					
	*Zostera noltii*	1	Netherlands	X	X	X	X		
			Germany	X	X	X	X		
		2	Mauritania	X	X	X			

*Climatic region was divided into temperate or tropical classifications and in some cases bioregions (Short et al., 2007). Temperate climates included bioregions 1 (Temperate Atlantic), 3 (Mediterranean), 4 (Temperate North Pacific) and 6 (Temperate Southern Ocean). Tropical climates included bioregions 2 (Tropical Atlantic) and 5 (Tropical Indo-Pacific)*.

* *FTIR analysis, NMR analysis*.

### Thermogravimetric analysis (TGA)

At UTS, all samples were analyzed using thermogravimetry (SDT Q600, TA Instruments, New Castle, DE, USA) with a 0.1 μg balance sensitivity. An aliquot of ground sample (10 mg) was placed in a platinum cup and heated under N_2_ (gas flow 100 mL min^−1^) at 10°C min^−1^ (Yang et al., 2006; Ncibi et al., 2009) to 600°C. Universal Analysis software (TA Instruments) was used to aid the identification and quantification of mass loss within specific temperature intervals. Delineation of thermal intervals (TI) was based on the rate-of-change derivative (% mass loss °C^−1^) which indicated distinct temperatures of mass loss (Yang et al., 2006, 2007). Briefly, mass loss occurring from 30 to 180°C was associated with the loss of moisture. The first temperature interval (TI_1_: labile; hemicellulose) ranged from 180 to 220°C, followed by TI_2_ (labile; hemicellulose) from 220 to 300°C. TI_3_ (refractory; cellulose) extended from 300 to 400°C, then TI_4_ (refractory; lignin and insoluble polysaccharide residues) from 400 to 600°C. TI_1−4_ were recalculated as a gravimetric proportion of total organic matter (OM) from 180 to 600°C, i.e., analogous to OM calculations via loss on ignition.

### Fourier-transform infrared (FTIR) spectroscopy

FTIR analyses were performed on a subset of untreated samples (Table 2) using FTIR instrumentation described in (Baldock et al., 2013b) (Table 2). Diffuse reflectance mid/near-infrared spectra were acquired over 6000–400 cm^−1^ with a resolution of 8 cm^−1^. The background signal intensity was quantified by collecting 240 scans on a silicon carbide disk before analyzing any samples. In total, 60 scans were acquired and averaged to produce a reflectance spectrum for each individual sample. The Omnic software (Version 8.0; Thermo Fisher Scientific Inc.) was used to convert the acquired reflectance spectra into absorbance spectra (log-transform of the inverse of reflectance).

Fourier-Transform Infrared (FTIR) spectra were pre-processed for statistical analysis using The Unscrambler 10 (Camo, Oslo) software package. Second derivatives were generated for the spectra in order to reveal signals hidden within broad peaks using the Savitsky-Golay function with nine smoothing points. Scattering effects were then removed using the Extended Multiplicative Scatter correction algorithm. A principal components analysis (PCA) model was then built using two-thirds of the data set (i.e., calibration set). The remaining one-third of the data was used for validation of the model. The PCA model revealed groupings within the data that would be otherwise hidden within the complex spectra. In using the second derivative for this model, the positive and negative loadings will be associated with the negative and positive regression coefficients, respectively. Lastly, peaks were identified from assignments for biological tissues (Movasaghi et al., 2007).

### Solid state ^13^C-CPMAS nuclear magnetic resonance (NMR) spectroscopy

A subset of samples (Table 2) analyzed with FTIR spectroscopy were also analyzed using solid-state ^13^C-Cross-Polarisation Magic-Angle Spinning (CPMAS) (NMR) spectroscopy (Baldock et al., 2013a, with slight modification). Briefly, a 200 Avance spectrometer (Bruker Corporation, Billerica, MA, USA) equipped with a 4.7 T, wide-bore superconducting magnet operating at a resonance frequency of 50.33 MHz was used to obtain the spectra. Weighed samples (200–400 mg) were packed into 7 mm diameter zirconia rotors with Kel-F® end caps and spun at 5 kHz. A cross-polarization ^13^C-NMR (CP) analysis using a 90° pulse of 3.2 μs at 195 W, a contact time of 1 ms, and a recycle time ranging from 1 to 5 s was used for all samples with a total of 5,000 scans being collected for each sample. The duration of the recycle delay was set to <5 times sample specific T_1_H values determined using an inversion recovery pulse sequence. All spectral processing was completed using the Bruker TopSpin 3.1 software (Baldock et al., 2013a). The acquired total signal intensity was divided into a series of chemical shift regions: amide/carboxyl/ketone (215–165 ppm), O-aromatic (165–145 ppm), aromatic (145–110 ppm), di-O-alkyl (110–95 ppm), O-alkyl (95–60 ppm), N-alkyl/methoxy (60–45 ppm), and alkyl (45–10 ppm). The NMR spectral intensities were entered into a molecular mixing model (MMM) for terrestrial soils (Baldock et al., 2004) in order to predict macromolecule content (carbohydrate, lignin, lipid and protein) of the seagrass tissue. The original terrestrial protein component of the MMM was altered to be reflective of the amino acid composition of seagrasses using data from *Posidonia australis, Thalassia testudinum, Halodule wrightii*, and *Syringodium filiforme* (Zieman et al., 1984; Torbatinejad et al., 2007). The model was constrained with the elemental carbon (C) and nitrogen (N) content of the tissues. Elemental C:N data was obtained from bulk tissue using an elemental analyzer (LECO TruSpec, MI, USA). If there was not enough material for CN analysis, estimates were obtained from the literature (Duarte, 1990; Hansen et al., 2000; Lavery et al., 2009).

### Assumptions and limitations for experimental design and analyses

Trends in climatic variation are likely strongly influenced by taxa since most genera are strictly found in either tropical or temperate regions. This confounding factor needs to be considered when making any general inferences between tropical and temperate seagrasses. Therefore, we chose to analyze the data in three ways in order to draw meaningful conclusions about the data within these limitations. First, both tropical and temperate groups of seagrass contain both small, ephemeral genera and large, persistent genera. While the number of samples obtained for the large taxa were higher for the tropical (9) compared to the temperate (3) samples due to limitations in collecting protected, endemic Australian *Posidonia*, we were otherwise satisfied that the range of morphology-based functional groups were represented in both climatic regions. Second, when we analyzed taxonomic variation in organic composition, we accounted for confounding factors by normalizing for climatic region and tissue type (i.e., genera nested within family, family nested in climatic region for each tissue type). Lastly, *Zostera* was the most well sampled genus and contained both temperate and tropical samples. We took advantage of this broad sampling and used *Zostera* as a case-study for climatic influence within a taxonomic group.

There are assumptions and limitations that also need to be noted for data interpretation of the thermogravimetric (TGA) and NMR (MMM) analyses used herein. For TGA, the interpretation of pyrolysis data of vascular plants has often been limited to lignocellulose content. Yet, as pyrolysis of protein- and lipid-rich microalgae have shown that these components can be lost at temperatures ≥300°C (Kapusniak and Siemion, 2007; Kebelmann et al., 2013); therefore, protein and lipids are likely mixed within the lignocellulose fraction of the seagrass TGA data. For the MMM, analyses were performed under the constraint of maintaining sample-specific C:N molar ratios. When run in this manner, the MMM has no option but to allocate all sample N to protein. In samples with appreciable content of inorganic N or non-protein forms of organic N, the allocation of C to protein within the MMM will be over-predicted. Additionally, an overestimation of lignin content from MMM is possible (Baldock et al., 2004). When comparing the NMR spectra to the MMM output, there can be an over-allocation to the N-Alkyl/Methoxyl region (60–45 ppm) suggesting the model is over-predicting the sum of lignin+protein. Although the model parameters were modified to fit the amino acid profile of seagrasses, the lignin component includes both gymnosperm and angiosperm lignin (Baldock et al., 2004). Since seagrasses are angiosperms, this may partly explain the poor relationship between the spectra and the MMM. Furthermore, seagrasses contain other compounds that include aromatics other than phenolics and lignin, such as suberin and chlorophyll (Enríquez et al., 1992; Papenbrock, 2012), which may further explain a deviation of the MMM from the spectra.

### Statistical analyses

Multi-factorial permutational analysis of variance (PERMANOVA) was used to investigate differences in the size of the four temperature intervals (TI_1_, TI_2_, TI_3_, TI_4_ normalized to total organic mass loss as well as total organic matter, OM) between taxa, tissue type and climatic zone for TGA and MMM analyses. Preliminary 1-factor analyses comparing TGA intervals for *tissue types* (6 levels: leaf, rhizome, root, sheath, stem, and vertical rhizome) identified that there were significant differences amongst most tissue types [*Pseudo-F*_(5, 145)_ = 9.75 *P-perm* = 0.001]. There was no significant difference between sheath, stem and vertical rhizome. Although they represent diversity in morphology, chlorophyll content and location across the water-sediment interface, they were combined into a tissue category that represented most of the samples: “non-photosynthetic above-ground” tissue. A 3-factor PERMANOVA further examined differences in pyrolysis dynamics for tissue types, independently, comparing *climatic zone* (temperate or tropical), *family nested within climatic zone* and *genus nested within family*. Since *Zostera* was the most well sampled taxonomic group, finer-scale variation in tissues and individual bioregions from the climatic zones (Temperate Atlantic, Tropical Atlantic, Mediterranean, Temperate North Pacific, Tropical Indo-Pacific, Temperate Southern Ocean) were investigated within the *Zostera* genus with a 2-way PERMANOVA (*tissue* and *bioregion*) (Short et al., 2007). For NMR/MMM analyses, data was not as deeply sampled as TGA, so *tissue type* was not separated for statistical analysis. A 3-way PERMANOVA was used to analyze macromolecular content between *tissues* and *climatic zone* and *family nested in climatic zone*.

Pairwise comparisons of tissue types, climatic zone, families and genera were made. Monte Carlo approximated *P*-values [*P(MC)*] were used to interpret comparisons with low numbers of unique permutations (i.e., <100) for all analyses. Similarity percentage (SIMPER) analysis was used to identify the contributions of each temperature interval or MMM component to observed differences amongst tissue types, climatic zone and taxa. Principal components analysis (PCA) plots provided explanation of variation linked to the factors. PCA plots were also made between the molecular mixing model output (MMM) and TGA thermal intervals. C:N ratios were not included since they were accounted for in the MMM. Only the data that were analyzed for both NMR and TGA were used. Analyses were based on untransformed data and Euclidean distance resemblance matrices calculated by PRIMER v6 for Windows (PRIMER-E; Clarke, 1993). Analyses were also performed using PRIMER v6 with PERMANOVA+ add on.

## Results

### Thermogravimetric analyses (TGA)

**Figure 2:**
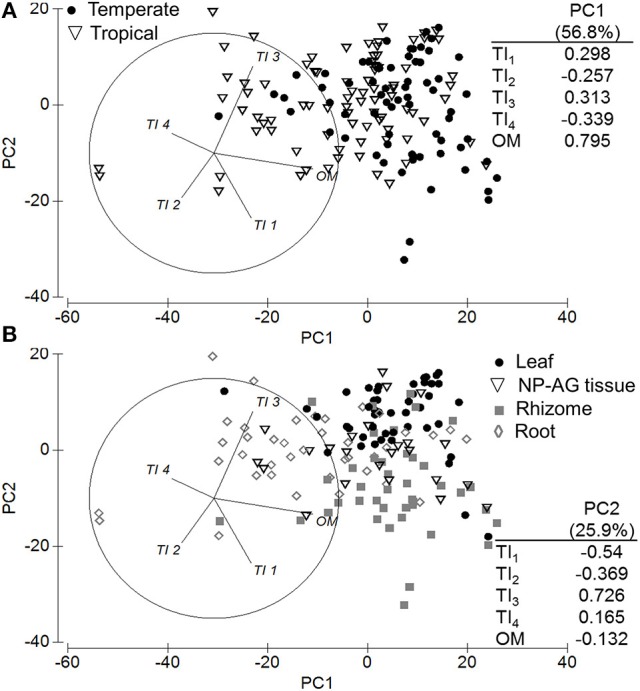
Principal components analysis and eigenvectors of seagrass organic matter quality using thermogravimetric analyses by **(A)** climatic zone and **(B)** tissue type (NP-AG = non-photosynthetic above-ground tissue). PC1 = 56.7% variation, PC2 = 25.6% variation. Thermal intervals (TI) represent distinct organic matter components from TGA normalized to total organic matter (TI_*1*_: labile, carbohydrates, hemicellulose, 180–220°C; TI_*2*_: labile, carbohydrates, hemicellulose, 220–300°C; TI_*3*_: refractory, cellulose, 300–400°C; TI_*4*_: refractory, lignin and residues, 400–600°C).

**Figure 3:**
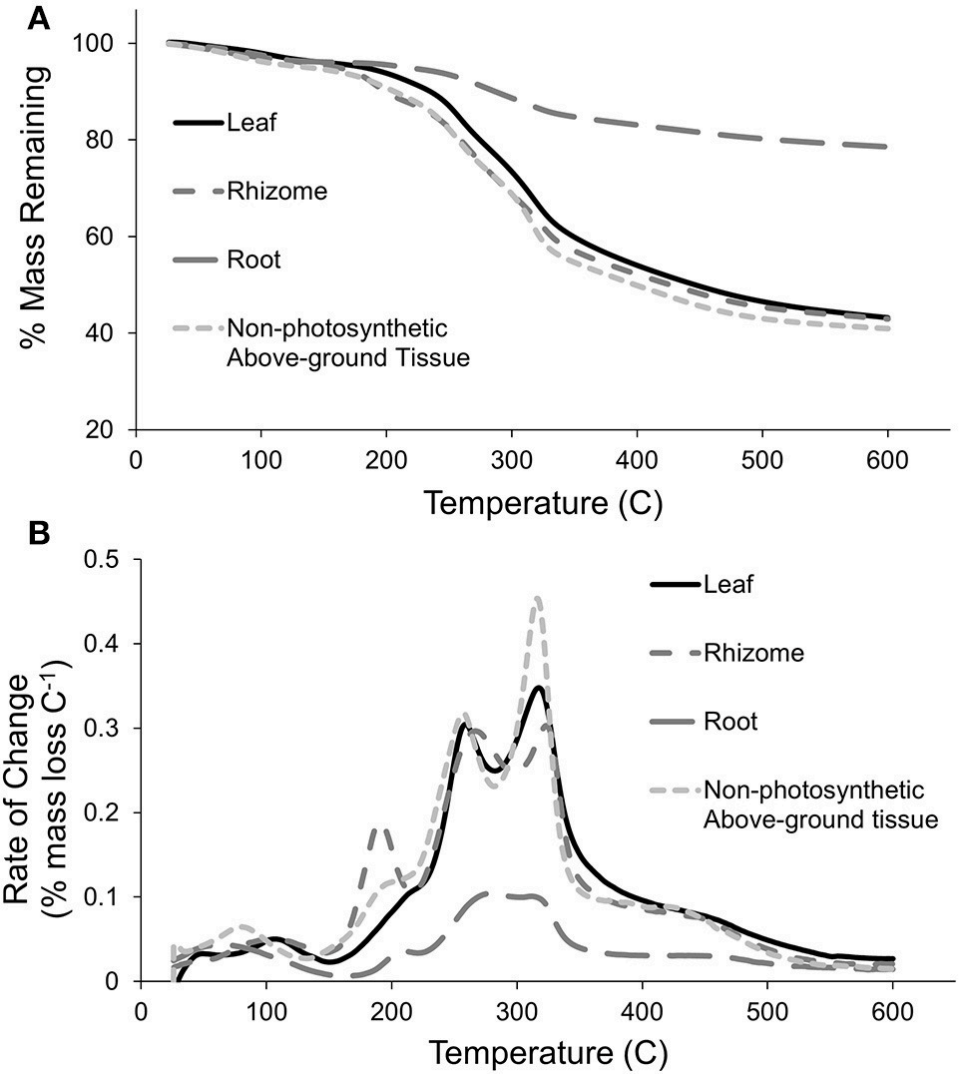
Representative thermograms for each tissue type, **(A)** % mass remaining with increasing temperature and **(B)** Derivative rate-of-change (% mass loss per °C) with increasing pyrolysis temperature.

**Figure 4:**
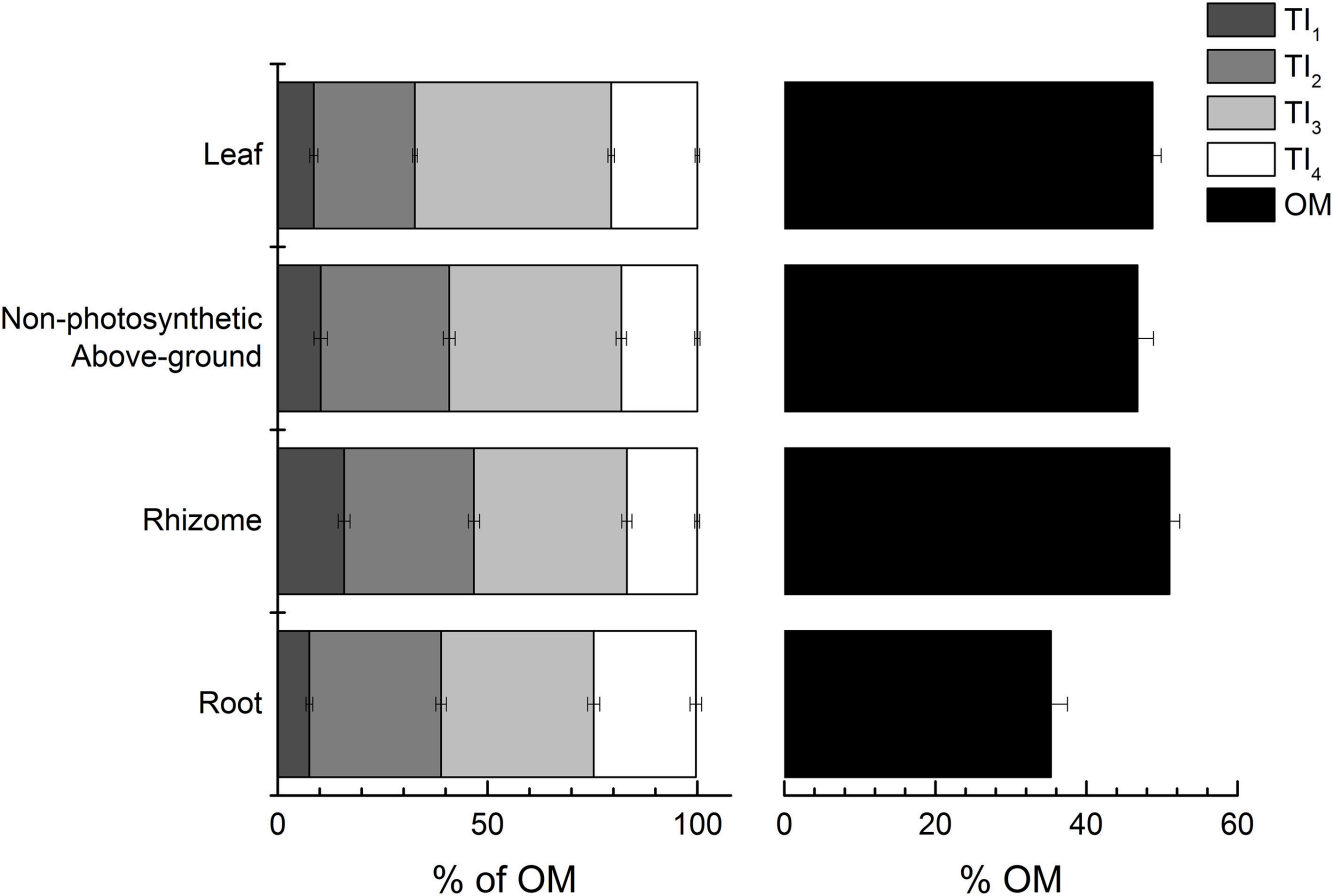
Thermal intervals (TI) as percent of total organic matter (OM) and OM as percent of the total mass across all tissue types. TIs represent distinct organic matter components from TGA (see Figure 2 for TI definitions). Values represent mean ± 1 S.E.M.

A PCA of all samples showed that climatic region driven by OM (56.7%) and tissue type driven by TI_3_ (25.6% of OM) explained most of the variation in TGA results across the seagrass samples as PC1 and PC2, respectively (Figure 2). Seagrass tissues were further investigated and were shown to be significantly different from each other [*Pseudo-F*_(3, 147)_ = 15.964 *P-perm* = 0.001; pairwise tests *P-perm* ≤ 0.018; Figures 2–4; SIMPER values in Table 3]. Leaf tissues had the highest amount of cellulose-associated refractory OM (TI_3_, ~47%), while the roots had the highest amount of lignin-associated refractory OM (TI_4_, ~24%) and lowest overall OM content (35%). Rhizomes were highest in overall OM content (51%) and proportion of labile OM (TI_1_, ~16%). Non-photosynthetic above-ground tissue quality was intermediate between leaf and rhizome tissues (Figures 2B, 4).

**Table 3:** SIMPER table of significant PERMANOVA pairwise comparisons of thermogravimetric analysis (TGA) and molecular mixing model (MMM).

**Pairwise Comparison**		**TGA**						**MMM**			
		**Avg. Sq. Dist**.	**OM**	**TI_1_**	**TI_2_**	**TI_3_**	**TI_4_**	**Avg. Sq. Dist**.	**carbohydrates**	**Lignin**	**Protein**
Tissue	Leaf - Rhizome	674	48.8–51.0 (20.2)	8.58–15.8 (25.8)	24.1–31.0 (19.2)	46.8–36.4 (28.7)		474	51.8–64.4 (45.7)	21.3–23.4 (12.2)	23.3–11.3 (39.6)
	Leaf - Root	930	48.8–35.3 (45.5)		24.1–31.4 (13.5)	46.8–36.4 (23.1)	20.5–24.3 (10.7)	443	51.8–56.0 (17.7)	21.3–30.4 (33.7)	23.3–10.8 (45.9)
	Rhizome - Root	1079	51.0–35.5 (46.4)	15.8–7.55 (15.8)	31.0–31.4 (11.9)	36.4–36.4 (12.6)	16.8–24.3 (13.3)				
	Leaf - NP	496	48.8–46.8 (33.4)	8.58–10.3 (21.1)	24.1–30.6 (20.3)	46.8–41.0 (19.6)		657	51.8–63.1 (37.3)	21.3–28.0 (19.7)	23.3–7.93 (41.6)
	Rhizome - NP	614	51.0–46.8 (31.2)	15.8–10.3 (27.4)	31.0–30.6 (18.6)	36.4–41.0 (18.7)					
	Root - NP	866	35.3–46.8 (48.0)	7.55–10.3 (10.4)	31.4–30.6 (12.2)	36.4–41.0 (15.7)	24.3–18.1 (13.8)				
Latitudinal Region											
Temperate-Tropical								384	55.7–59.9 (37.9)	25.4–24.8 (27.44)	16.7–13.0 (32.45)
	Leaf	371	52.9–45.4 (38.7)	11.9–5.94 (29.7)	22.4–25.5 (8.47)	46.7–46.8 (15.3)					
	Rhizome	649	54.6–48.5 (23.9)	20.6–12.4 (30.4)	28.8–32.5 (22.9)	35.4–37.2 (18.4)					
	Root	912	41.4–31.1 (45.6)		31.5–31.3 (11.6)	41.1–33.0 (19.6)	19.6–27.7 (17.9)				
	NP	643	51.9–40.7 (42.5)	14.2–5.61 (23.3)	26.9–35.0 (19.4)	41.8–40.0 (11.7)					
Taxa: Family											
Hydrocharitaceae - Zosteraceae	NP (Temp.)	417	45.0–54.4 (27.7)	10.6–15.7 (21.1)	34.5–26.0 (20.9)	33.3–42.0 (23.0)					
Posidoniaceae - Zosteraceae	NP (Temp.)	493	43.6–54.4 (31.3)	4.36–15.7 (38.9)	30.1–26.0 (12.5)	45.7–42.0 (14.4)					
Cymodoceaceae - Posidoniaceae	(Temp.)							454	58.3–55.3 (12.2)	17.4–30.8 (48.3)	23.0–12.4 (38.7)
Hydrocharitaceae - Cymodoceaceae	(Trop.)							467	61.8–57.4 (40.1)	21.1–29.5 (33.6)	15.4–10.2 (24.3)
Taxa: Genus											
*Enhalus-Thalassia*	NP	746	44.6–32.5 (23.4)		25.9–40.9 (34.2)	47.3–31.2 (39.0)					
*Zostera*: Tissue											
	Leaf - Rhizome	758	50.7–52.4 (14.7)	11.0–18.3 (28.5)	24.3–32.2 (22.0)	45.5–34.4 (31.0)					
	Leaf - Root	660	50.7–38.2 (47.9)	11.0–6.01 (15.8)	24.3–34.1 (18.0)	45.5–39.6 (15.1)					
	Rhizome - Root	939	52.4–38.2 (42.1)	18.3–6.01 (26.1)	32.2–34.1 (11.5)	34.4–39.6 (15.6)					
	Root - NP	633	38.2–53.4 (59.4)	6.01–13.7 (15.7)	34.1–27.2 (9.87)	39.6–42.6 (10.3)					
*Zostera*: Bioregion	Temp. Atl. - Trop. Atl.	827	51.8–36.2 (41.7)	14.3–9.14 (17.0)	27.6–37.3 (18.5)	41.2–33.1 (18.8)					
	Temp. Atl.- Indo-Pacific	668	51.8–40.0 (32.4)	14.3–8.78 (21.3)	27.6–37.3 (22.7)	41.2–34.4 (20.3)					
	Trop. Atl. - Temp. Pacific	489	36.2–44.6 (35.7)	9.14–10.4 (12.0)	37.3–29.7 (21.8)	33.1–41.6 (26.0)					
	Temp. Pacific - Indo-Pacific	383	44.6–40.0 (26.2)	10.4–8.78 (14.9)	29.7–37.3 (27.6)	41.6–34.4 (27.9)					

*Values represent average abundance of each variable followed by the percent contribution in parentheses. NP, Non-photosynthetic above-ground tissue; Temp, temperate; Trop, tropical; Atl, Atlantic. Thermal intervals (TI) represent distinct organic matter components from TGA normalized to total organic matter (TI_1_: labile, carbohydrates, hemicellulose, 180–220°C; TI_2_: labile, carbohydrates, hemicellulose, 220–300°C; TI_3_: refractory, cellulose, 300–400°C; TI_4_: refractory, lignin and residues, 400–600°C)*.

**Figure 5:**
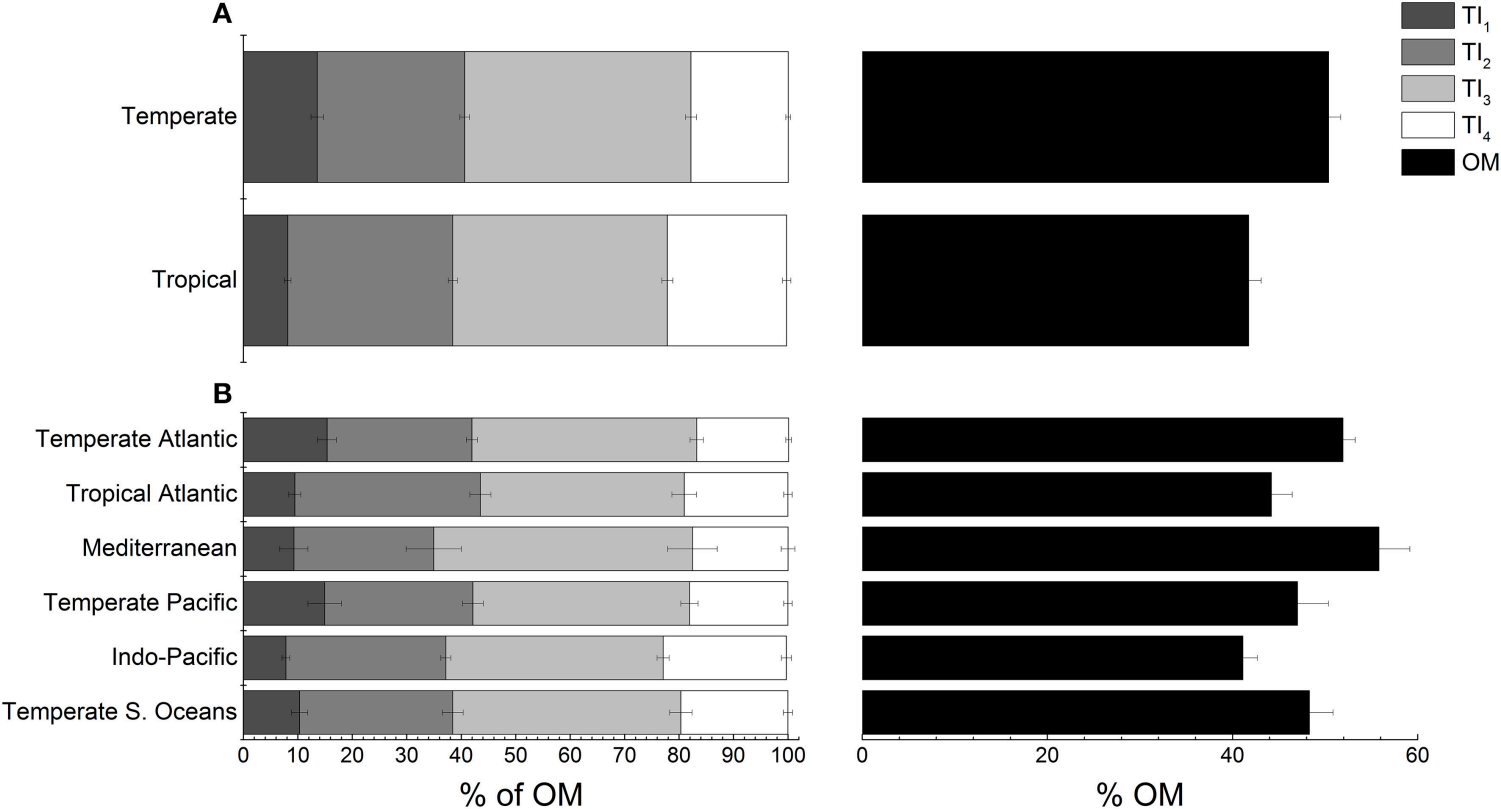
Seagrass organic matter quality for all tissues types from thermogravimetric analyses between **(A)** temperate and tropical regions and **(B)** across all bioregions. Thermal intervals (TI) represent distinct organic matter components from TGA (see Figure 2 for TI definitions). Values represent mean ± 1 S.E.M.

Investigation of the variation in climatic zone and taxa for each tissue type revealed that differences in temperate and tropical TGA signatures were consistent and explained most of the variation across all tissue types, which was driven by OM content (PC1 = 46.2–70.1%; Figure 5; Table 3). Temperate regions had highest total OM in all tissue types (Figure 5; Table 3). For leaf [*Pseudo-F*_(1, 32)_ = 7.01 *P-perm* = 0.006], rhizome [*Pseudo-F*_(1, 27)_ = 3.65, *P-perm* = 0.015] and non-photosynthetic above-ground tissues [*Pseudo-F*_(1, 13)_ = 5.26, *P-perm* = 0.009], labile TI_1_ content was 1.5–2.5-times higher than their tropical counterparts (Table 3). Refractory OM content was higher in the tropics for leaf and rhizome tissues as TI_3_, while roots [*Pseudo-F*_(1, 26)_ = 3.44, *P-perm* = 0.048] had higher TI_4_, but lower TI_3_ in the tropics (Table 3).

**Figure 6:**
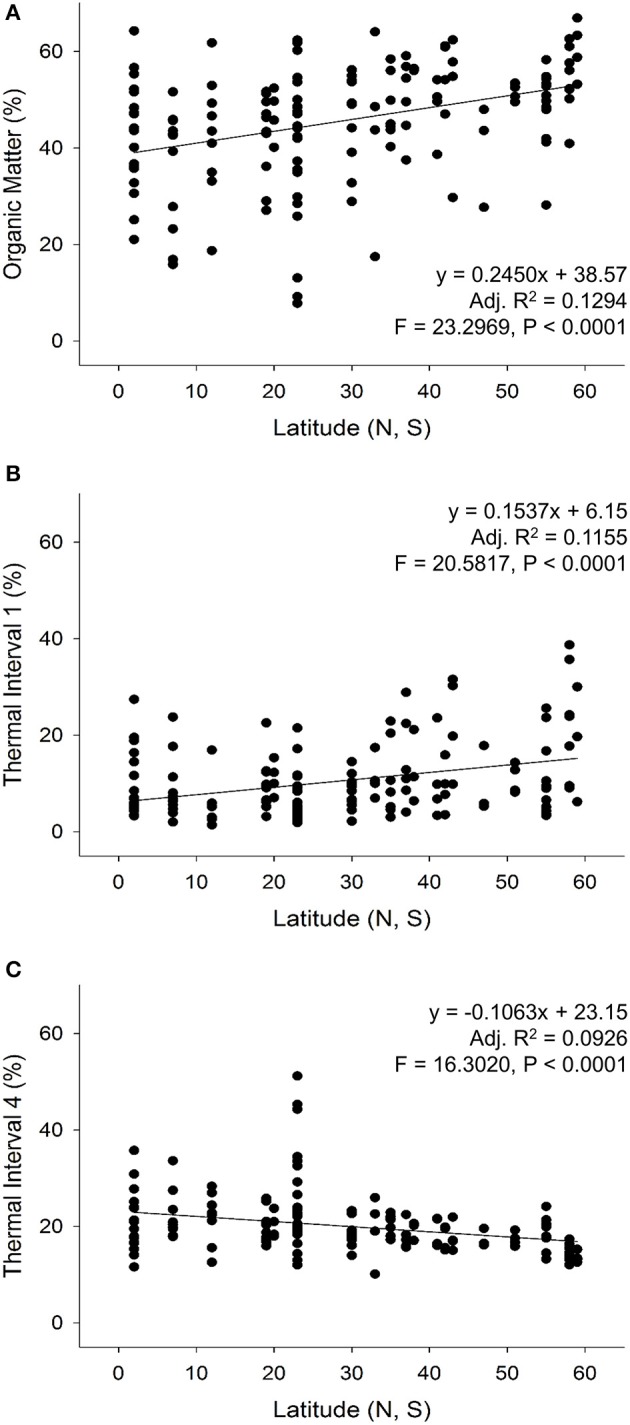
Linear regressions of the strongest relationships (adjusted *R*^2^ > 0.09) between TGA **(A)** organic matter, **(B)** thermal interval 1, and **(C)** thermal interval 4 with latitudes for all tissue types.

Linear regressions of OM and the thermal intervals from all tissues across latitudes revealed that increased % OM content had a strong, highly significant positive relationship with higher latitudes (Adj. *R*^2^ = 0.1294, *F* = 23.2969, *P* < 0.0001; Figure 6A). The first three temperature intervals showed a similar positive relationship, which was highly significant for TI_1_ (Adj. *R*^2^ = 0.1155, *F* = 20.5817, *P* < 0.0001; Figure 6B), a weaker, but significant relationship with TI_2_ (Adj. *R*^2^ = 0.0280, *F* = 5.3256, *P* < 0.0224) and an insignificant relationship for TI_3_ (Adj. *R*^2^ < 0.001, *F* = 0.8782, *P* = 0.3502). Even though lignin-associated (TI_4_) content did not contribute to similarities in SIMPER analysis (Table 3), TI_4_ content had a highly significant inverse relationship with increasing latitude (Adj. *R*^2^ = 0.0926, *F* = 16.3020, *P* < 0.0001; Figure 6C). When northern and southern latitudes were compared across OM and TIs separately, the OM and TI_4_ relationship became 1.5–2-times stronger and highly significant for the Northern Hemisphere samples (Adj. *R*^2^ = 0.1891, *F* = 23.8478, *P* < 0.0001 and Adj. *R*^2^ = 0.1829, *F* = 22.9296, *P* < 0.0001, respectively). Conversely, the OM and TI_4_ relationship within the Southern Hemisphere samples were insignificant (Adj. *R*^2^ = 0.0266, *F* = 2.3942, *P* = 0.1281 and Adj. *R*^2^ = 0.0061, *F* = 1.3114, *P* = 0.3576, respectively), while the positive, significant relationship between latitude and TI_1_ was maintained (Adj. *R*^2^ = 0.1378, *F* = 9.1508, *P* = 0.0039).

**Figure 7:**
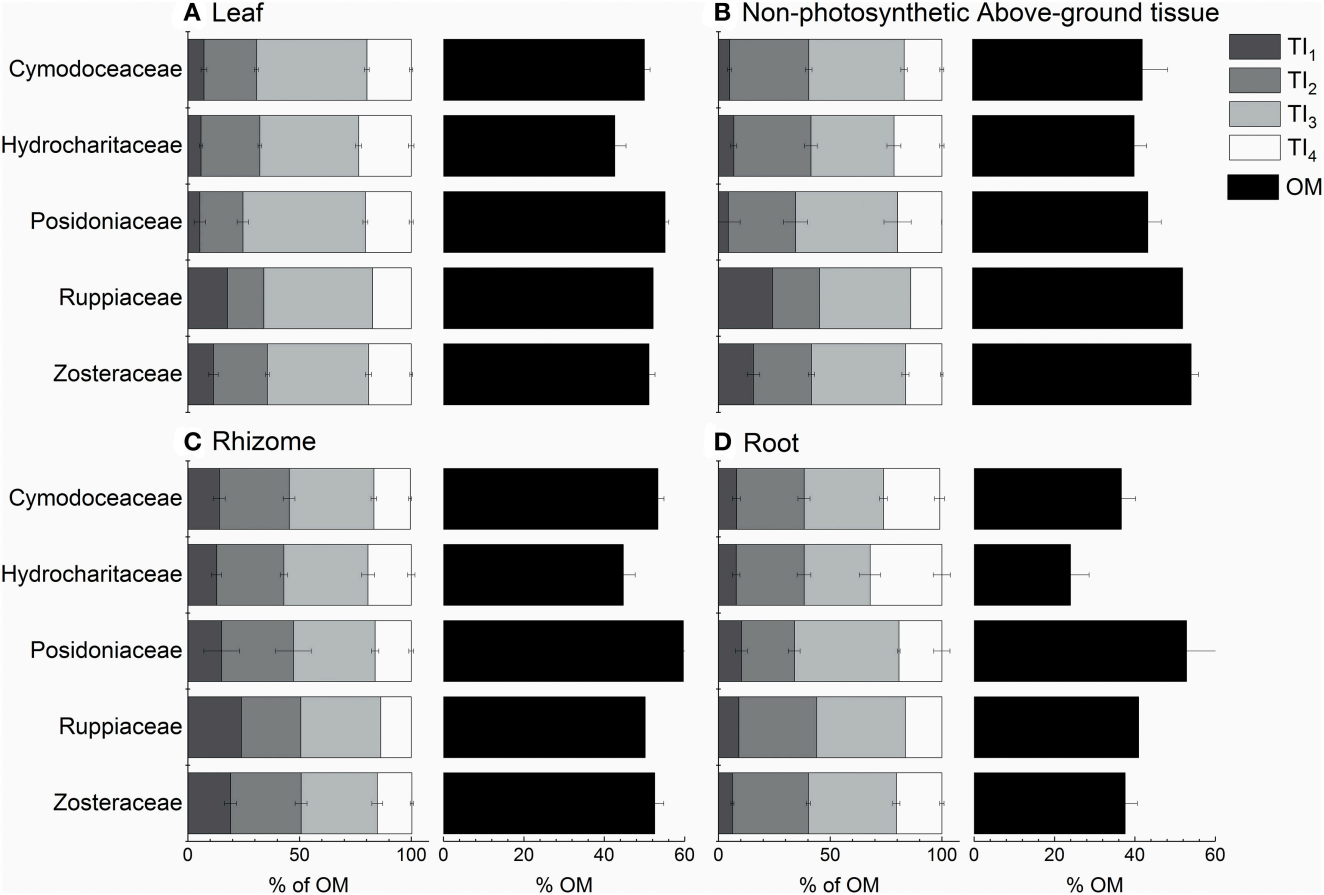
Seagrass organic matter quality from thermogravimetric analyses across families for **(A)** leaf, **(B)** non-photosynthetic above-ground tissue, **(C)** rhizome, and **(D)** root tissues. Thermal intervals (TI) represent distinct organic matter components from TGA (see Figure 2 for TI definitions). Values represent mean ± 1 S.E.M.

There was no influence of taxa on leaf, rhizome or root thermograms (taxa nested within each tissue type; Figure 7; Table 3). Non-photosynthetic above-ground tissues had significant differences among families [*Pseudo-F*_(4, 13)_ = 3.05, *P-perm* = 0.017] and genera [*Pseudo-F*_(5, 13)_ = 3.76, *P-perm* = 0.007]. Pairwise tests showed that significant differences between temperate Posidoniaceae and Zosteraceae (*t* = 2.83, *P-perm* = 0.010) were driven by a higher amount of OM and TI_1_ in Zosteraceae. Temperate Hydrocharitaceae and Zosteraceae were also significantly different [*t* = 2.15, *P(MC)* = 0.034], driven equally by higher OM, TI_1_ and TI_3_ content in Zosteraceae. Within the tropical Hydrocharitaceae family, *Enhalus* contained higher TI_3_ and OM than *Thalassia*, which was higher in TI_2_ [*t* = 2.91, *P(MC)* = 0.045].

**Figure 8:**
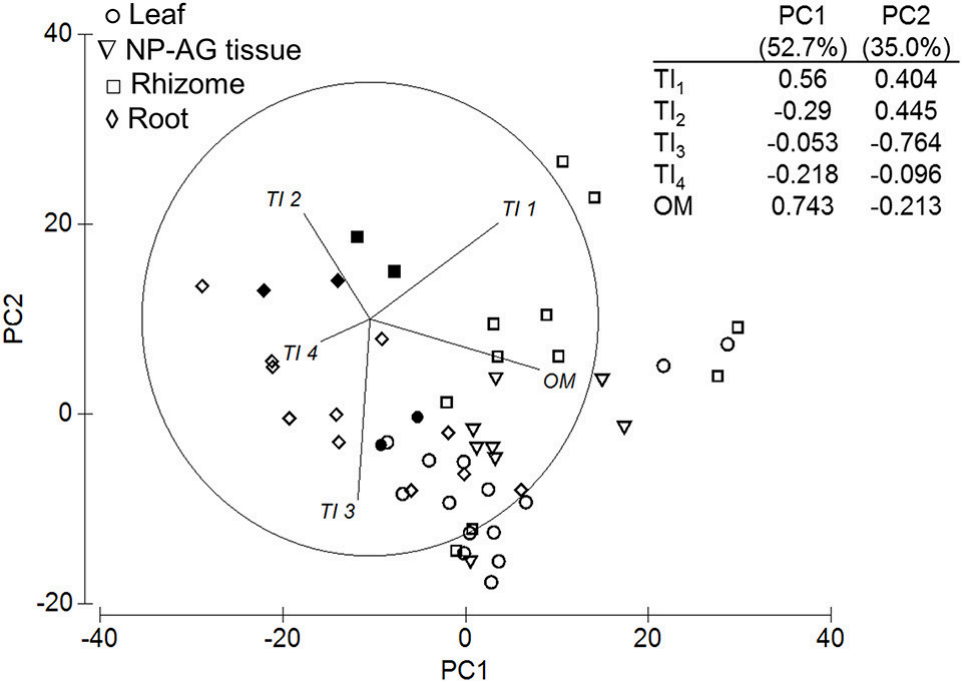
Principal components analysis and eigenvectors of TGA for *Zostera* samples. Open symbols represent temperate seagrass and solid symbols represent tropical seagrass. PC1 = 52.7% variation, PC2 = 35.0% variation. NP-AG = non-photosynthetic above-ground tissue.

Within-*Zostera* analyses showed similar trends as the overall tissue [*Pseudo-F*_(3, 32)_ = 7.52, *P-perm* = 0.001] and climatic region analyses [*Pseudo-F*_(4, 32)_ = 2.29, *P-perm* = 0.026], e.g., OM drove the 52.7% variation in PC1, while TI_3_ drove 35.0% variation in PC2 (Figure 8; Table 3). Non-photosynthetic above-ground tissues were not significantly different between leaves and rhizomes, and TI_4_ did not contribute to the differences among tissue types. Instead, roots contained a higher proportion of TI_2_, and rhizomes contained higher OM and TI_1_ than leaves (Figure 8). Temperate Atlantic and Pacific *Zostera* species had consistently more OM and higher proportions of TI_3_ and TI_1_, while Tropical Atlantic and the Indo-Pacific *Zostera* had more TI_2_ (Table 3). *Zostera* samples from the Temperate Southern Ocean were not significantly different from the other four bioregions.

### NMR molecular mixing model (MMM)

The NMR spectral intensity regions of each tissue type are summarized in Table S2. The 4-component model (carbohydrate, protein, lignin, lipid) was chosen to predict the macromolecular content of the seagrasses. The root mean squared error (RMSE) of this model was higher than the 5-component model that included a pure carbonyl component (1.8–4.5 vs. 0.8–1.5). However, the carbonyl component typically represents a decomposition by-product, which would not be present in fresh samples.

**Figure 9:**
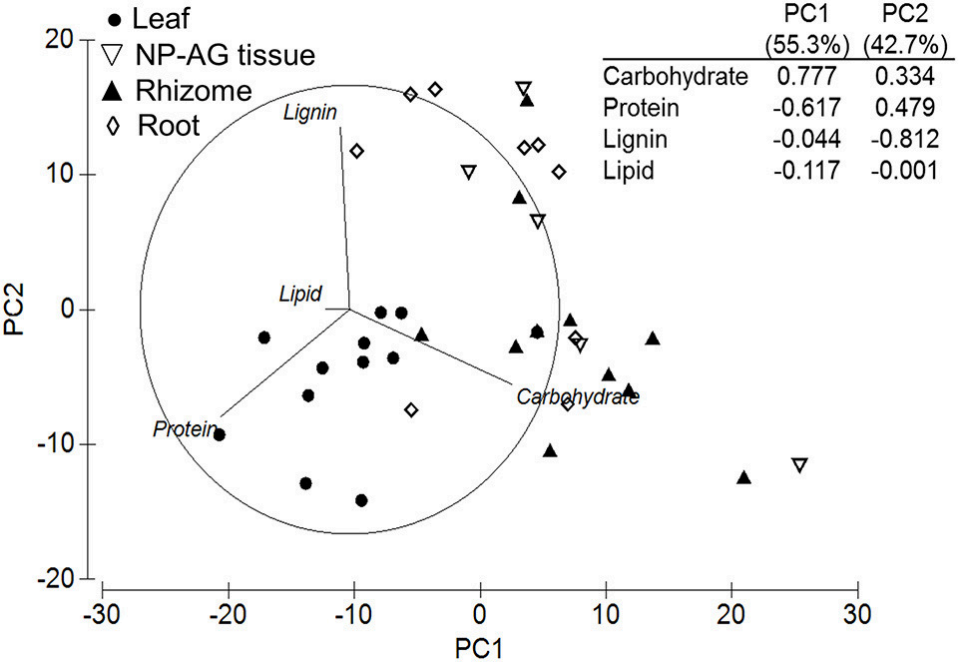
Principal components analysis and eigenvectors of molecular mixing model predictions from NMR analysis. PC1 = 55.3% variation, PC2 = 42.7% variation. NP-AG = non-photosynthetic above-ground tissue.

PCA analysis revealed that both PC1 (55.3%) and PC2 (42.7%) were explained by variation in tissues (Figure 9). PC1 was due to variation between protein-dominated leaves and carbohydrate-rich below-ground tissues. The variation in PC2 was driven by lignin-rich roots. Non-photosynthetic above-ground tissue data points were diffuse across PC2 (Figure 9). PERMANOVA analysis revealed that the significantly different macromolecular content across tissue types [*Pseudo-F*_(3, 15)_ = 6.09, *P-perm* = 0.001; Figure 9; Table 3] was driven by differences between leaves and non-photosynthetic above-ground tissues (*t* = 4.47, *P-perm* = 0.001), leaves and rhizomes (*t* = 3.69, *P-perm* = 0.002), and leaves and roots (*t* = 2.71, *P-perm* = 0.01). SIMPER analysis showed that lignin content was highest in roots (30.4%) and lowest in leaf tissues (21.3%). Protein was highest in leaves (23.3%) and very low in non-photosynthetic above-ground tissues (7.9%) and root tissues (10.8%). Carbohydrate content was highest in rhizome tissues (64.4%).

There were marginally significant differences between temperate and tropical MMM predictions [*Pseudo-F*_(1, 15)_ = 3.03, *P-perm* = 0.052; Table 3], primarily due to higher carbohydrate content in the tropical samples and more protein in the temperate samples. Lignin content was similar between climatic zones, though tropical samples contained slightly higher aromatic content (Table 3). Additionally, significant differences were found between a few seagrass families [*Pseudo-F*_(5, 15)_ = 2.95, *P-perm* = 0.009]: tropical Hydrocharitaceae and Cymodoceaceae (*t* = 2.18, *P-perm* = 0.021) and temperate Cymodoceaceae and Posidoniaceae (*t* = 3.09, *P-perm* = 0.006). The differences in the two tropical families were driven by higher carbohydrate and protein content in Hydrocharitaceae and higher lignin content in Cymodoceaceae (Table 3). Alternatively, temperate Cymodoceaceae contained more protein and slightly more carbohydrates than Posidoniaceae, which had higher lignin content (Table 3).

### FTIR spectroscopy

**Figure 10:**
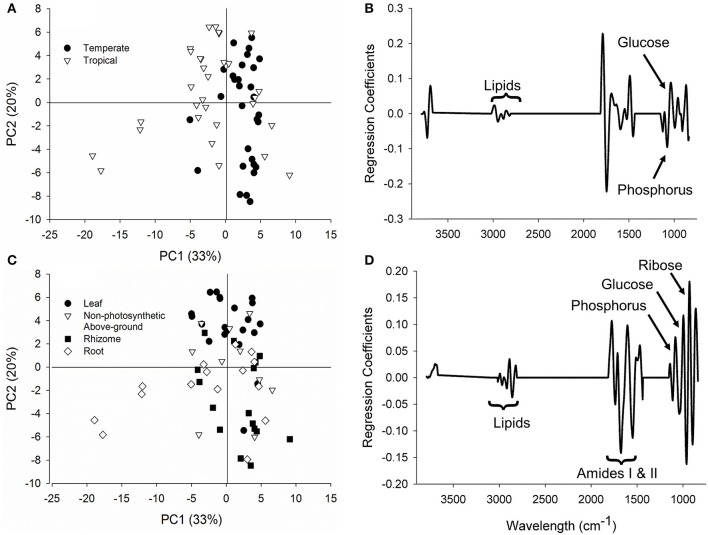
FTIR spectroscopy loadings and PCA scores of the seagrass samples. **(A,B)** Climatic zone (PC1 = 33% variation) where positive regressions coefficients correspond with greater signals in tropical regions and negative coefficients with temperate regions. **(C,D)** Tissue type (PC2 = 20% variation) where positive regressions coefficients correspond with greater signals in rhizomes and roots and negative coefficients with leaves.

Climatic zone explained most of the variation in FTIR data (PC1 = 33%), followed by tissue type that explained a further 20% of the variation (PC2; Figure 10). PC1 was driven by tropical seagrasses having higher lipid (~2,985 cm^−1^, lipid carbonyl stretching) and glucose (~1,040 cm^−1^), while temperate seagrasses had more phosphorus (~1,080 cm^−1^, P = O stretching) content (Figures 10A,B). PC2 loadings showed that leaf tissues had higher lipid content as lipid carbonyl stretching (~2,860 cm^−1^) as well as protein content indicated by higher Amide I (~1,670 cm^−1^) and Amide II (~1,560 cm^−1^) levels (Figures 10C,D). Rhizome and root tissues were characterized by higher glucose and ribose (1,020–995 cm^−1^) and phosphorus levels (~1,080 cm^−1^) (Figures 10C,D). Cellulose content (~1,150 cm^−1^) did not vary greatly among tissue types. Non-photosynthetic above-ground tissues showed intermediate biochemical qualities between leaf and rhizome tissues (Figure 10C).

### Comparison of MMM and TGA data

**Figure 11:**
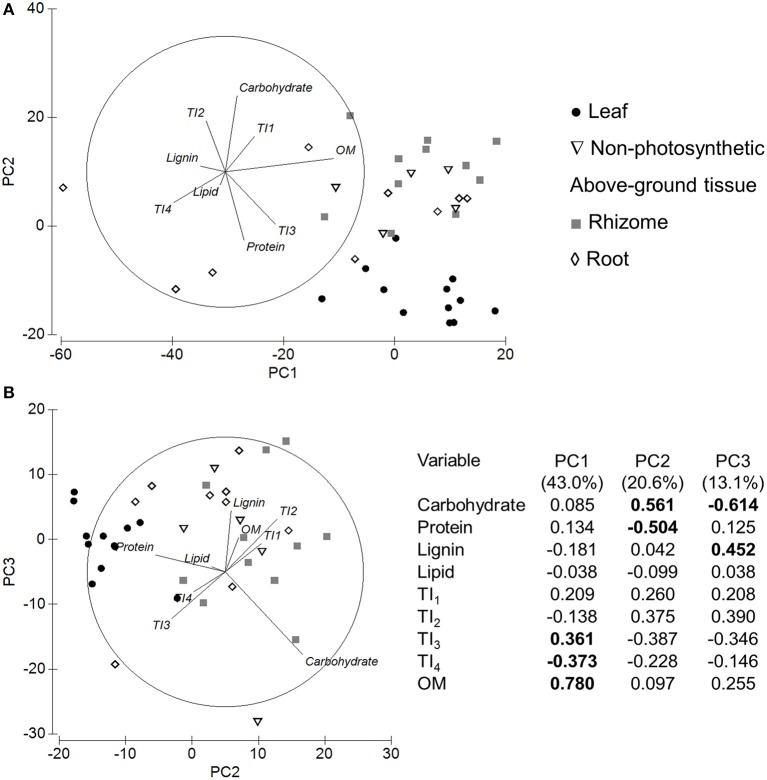
PCA plots for both NMR molecular mixing model and thermogravimetry variables. Plot **(A)** shows PC1 and PC2, and plot **(B)** shows PC2 and PC3. The table represents the Eigenvalues and Eigenvectors for each PC and variable. The highest correlations in each PC are bolded. Samples are presented as tissue type.

A PCA was used to compare the NMR (MMM) signatures with the TGA thermal intervals (39 samples total; Figure 11). The variability in PC1 (43.0%) was driven by higher OM in most samples, while the high TI_4_ content was driven by three root samples. PC2 (20.6%) was driven by higher protein in the leaf tissues and carbohydrate content in the rhizomes. Along the PC1 and PC2 axes, the orientation of protein and TI_3_ were similar, while lignin was orientated between TI_4_ and TI_2_ (Figure 11A). PC3 (13.1%) variation was driven by lignin and carbohydrate content (Figure 11B). The lignin looked to be linked to root and rhizome tissues, however, plotting of the variables by genus (data not shown), suggesting *Posidonia* and *Thalassodendron* were driving the higher lignin content. The carbohydrate content of *Enhalus* was driving the negative axis if PC3.

### Comparison of macromolecular content with previous literature values

The three non-destructive analyses used to assess the macromolecular content of seagrass tissues provided quantification of carbohydrates and lipids in-line with previous literature reports, but in some cases had overestimations of protein and lignin content. Lipid concentrations were highest in leaf tissues (Figures 9, 10) and corresponded with previously reported concentrations for all tissues (≤4% DW) (Lowe and Lawrence, 1976; Dawes and Lawrence, 1980, 1983; Lawrence et al., 1989; Dawes and Guiry, 1992; Touchette and Burkholder, 2002). Carbohydrates made up more than half of the macromolecule content of seagrasses and likely represented both soluble (oligosaccharide, starch, hemicellulose; Dubois et al., 1956) and insoluble (remainder by subtraction, e.g., cellulose) carbohydrate content. Studies that reported both soluble and insoluble carbohydrate content of *Zostera, Thalassia* and *Halophila* plants reported very similar ranges of carbohydrate content to this study for leaves (38–60% DW), rhizomes (54–70% DW), and roots (30–60% DW) (Dawes and Lawrence, 1983; Dawes et al., 1987; Dawes and Guiry, 1992). Data on sheath and stem components are not as common, but reported values from *Thalassia* and *Posidonia* coincided with carbohydrate (31–70% DW) and protein (2.5–10% DW) concentrations as found in this study (Table S1; Dawes and Lawrence, 1983; Lawrence et al., 1989).

The average leaf protein content (23%) predicted by MMM was higher than most studies have recorded (10–12% DW), although some have reported concentrations upwards of 20% DW (Dawes and Lawrence, 1979, 1980, 1983; Dawes et al., 1987; Dawes and Guiry, 1992; Siegal-Willott et al., 2010). The potential over-estimation of protein may have resulted from (a) previous underestimation of amino acid or protein content from wet chemistry methodologies that use strong chemicals to break apart molecules (e.g., Zieman et al., 1984; Lawrence et al., 1989), or (b) performing the MMM analyses under the constraint of maintaining sample-specific C:N molar ratios (see assumptions in section Assumptions and Limitations for Experimental Design and Analyses). In samples with appreciable contents of inorganic N or non-protein forms of organic N, the allocation of C to protein within the MMM will be over-predicted. Conversely to the leaf tissues, rhizome and root protein content in this study was comparable to the literature (4–17 and 4–7%, respectively) (Dawes and Lawrence, 1979, 1980, 1983; Dawes et al., 1987; Dawes and Guiry, 1992). Both above- and below-ground seagrass tissues take up inorganic N from the water-column and sediments, respectively (Touchette and Burkholder, 2000). Unless samples were collected from areas of high nutrient loading or enrichment, most of the N in the tissues in this study would be expected to be in primarily organic forms.

The non-invasive, non-destructive solid-state NMR method and subsequent mixing model predicted much higher lignin concentration for all tissues (21–30%) compared to previously reported values, which were, on average, ~13% DW for any given tissue (Table 1), and only occurred at such high levels in a few individual reports for *Posidonia* and *Cymodocea* (Table S1; Lawler et al., 2006; Ncibi et al., 2009; Bettaie et al., 2015). This could be a result of one or a combination of methodology effects: (a) an underestimation from using techniques that rely on chemical cell wall modification, and could result in losing material in the process (Hatfield and Fukushima, 2005) and (b) an overestimation of lignin content from MMM (see assumptions in section Assumptions and Limitations for Experimental Design and Analyses).

Rhizomes had the highest OM content which was dominated by labile OM from TI_1_ (Figure 4), which is contradictory with our hypothesis and previous reports of rhizome tissues having refractory components. Since rhizomes contain the energy reserves for seagrasses, the abundance of labile OM is likely due to storage of simple carbohydrates, and is potentially masking the relatively large lignocellulose content from TI_3_ and TI_4_ that would be expected in these rigid tissues. More than a quarter of the mass of pure sucrose is lost before 275°C in thermogravimetric analyses (Eggleston et al., 1996), which links with the large mass loss of sugars between 150 and 220°C for the rhizome tissues (Figure 3). In contrast to the rhizomes, roots had the least amount of total OM, with more than half the OM content as refractory OM (TI_3_ and TI_4_; Figure 4). The significantly higher proportion of TI_4_ coincides with significantly higher lignin content predicted by the MMM compared to the other tissues, although this was depicted in only 3 of the 10 root samples analyzed by NMR (Figure 11A). As hypothesized, increased lignin content would support better anchorage into sediments (Barnabas, 1991), but also could be involved in protection against microorganism invasions or water-logging (Kuo and Cambridge, 1978). Interestingly, FTIR analysis indicated that both rhizome and root tissues also had higher amounts of phosphorus compared to leaves (Figure 10). Rhizomes and roots do take up inorganic P from sediment porewater (Touchette and Burkholder, 2000), and so this higher P signal may represent this stored or in-transit inorganic P in the below-ground tissues.

Variation among taxa was not consistent across families (grouped within climatic zones) or genera (grouped within families) for NMR or TGA analyses. MMM analysis inclusive of all tissue types by ^13^C-solid state NMR revealed that there was significantly higher lignin content in tropical Cymodoceaceae than tropical Hydrocharitaceae (Table 3). This observation is in line with the previous reports of lignin content in these families (review in Table S1). The MMM predicted lower lignin content in temperate Cymodoceaceae compared to temperate Posidoniaceae (Table 3). While Posidoniaceae had the highest lignin content across all temperate seagrasses and tissue types (mean of 22.2% DW; Table S1), this study is the first to report lignin content for temperate Cymodoceaceae seagrasses.

## Discussion

### Seagrass tissue biochemistry

Tissue type explained nearly all of the variation in macromolecular content predicted by the NMR mixing model (MMM) and explained 26% of the variation in TGA data. Comparing both analyses together, the data suggested that (1) the carbohydrate content was dominated by soluble (labile) carbohydrates and hemicelluloses indicated by similar co-ordination with TI_1_ and TI_2_, and (2) there may be protein in TI_3_ (300–400°C), although TI_3_ is typically defined as cellulose in most thermal analysis studies (Figure 11; Yang et al., 2006; Wang et al., 2007). Such studies on vascular plants are often linked to biochar or biofuel applications, which are assessing lignocellulose content of plant residues or by-products (Yang et al., 2006; Carrier et al., 2011). Therefore, combining these two analyses provides insight into where the non-fiber macromolecules can be found in a thermogram.

### Climatic and taxonomic variation in seagrass organic composition

The location of seagrasses, whether temperate or tropical, had a strong influence on TGA thermograms and FTIR spectra when all samples were analyzed together. The influence of climatic region was also maintained once tissue types were separated for statistical analysis and when analyzing the *Zostera* genus alone. Temperate seagrasses were characterized by higher OM content, primarily as TI_1_ but also TI_3_, across all tissue types, with a positive correlation between OM and higher latitudes. This suggests that temperate seagrasses may, in general, have more labile storage carbohydrates in their tissues compared to tropical seagrasses. This is consistent with increasing biomass and production of above-ground tissues with increasing latitude found by Duarte and Chiscano (1999), although no latitudinal trends were previously found with below-ground biomass and production (Duarte and Chiscano, 1999). Additionally, the greater occurrences of nutrient limitations and grazing of tropical seagrasses may limit their biomass and production (Duarte and Chiscano, 1999). Furthermore, grazing has been shown to be a strong influence on carbohydrate reserves in both above- and below-ground tissues due to the export of soluble carbohydrates to support new leaf growth (Moran and Bjorndal, 2007; Eklöf et al., 2008). While the grazing pressures and nutrients regimes of the samples collected in this study are unknown or at best can only be estimated, the greater occurrences of nutrient limitations and grazing of tropical seagrasses may limit their biomass and production (Duarte and Chiscano, 1999).

Thermogravimetry showed that the total OM content of the seagrasses was variable across taxa for each tissue type, and thus would be useful for predicting total organic C content of seagrass species, e.g., Posidoniaceae had the highest OM content for leaves, rhizomes and roots (Figure 7). However, when the four OM fractions, normalized to total OM, were compared, the within-tissue type comparison revealed that taxa did not significantly influence the OM quality in leaf, rhizome or root tissues (Table 3). This could suggest that while some taxa may be more morphologically robust and have more above- and below-ground biomass than others, the overall OM quality is consistent across all seagrass taxonomic groups included in this study. Alternatively, it is possible that the limited number of replicates at the species level and the high morphological diversity within a family may have masked the differences in OM quality that we would have expected among species. TGA has been shown to be useful for identifying differences in OM quality across different phyla (macroalgae vs. vascular plants) and across orders of vascular plants (Trevathan-Tackett et al., 2015). As TGA is inexpensive and relatively quick, further studies on within-genera and within-species variability in OM quality would be suggested to reveal the finer taxonomic resolution of seagrass OM quality.

OM quality of non-photosynthetic above-ground tissues had the most variation across taxa (Figure 7B, Table 3). Most of this variation was revealed as greater refractory OM (TI_3_ and TI_4_) between temperate seagrass families (Posidoniaceae > Zosteraceae) and tropical Hydrocharitaceae families (*Enhalus* > *Thalassia*). These results indicate that sheath and stem biomass can significantly vary across taxa, and that more morphologically robust taxa typically have higher refractory OM content. The diversity in OM quality of the sheaths may also be related to their protective function of the leaf meristem and new shoots (Hemminga and Duarte, 2000; Kuo and den Hartog, 2006). This protective function could be more important for the morphologically robust taxa are *K*-strategists characterized by slower production and turnover rates (Duarte and Chiscano, 1999).

### Potential seagrass contribution to blue carbon stocks

The morphological and phylogenetic diversity among seagrasses has been hypothesized to influence the ability of some seagrasses to contribute to C stocks, i.e., their role as C donors (Macreadie et al., 2014). Specifically, the quality of organic C can influence the remineralization by microbes, which relies both on stoichiometry (C:N:P:S ratios) and production of specific exo-enzymes by microbes and decomposers for the breakdown of complex C, such as lignin (Enríquez et al., 1993; Couteaux et al., 1995; Silver and Miya, 2001; Sinsabaugh et al., 2002). It is likely that nutrient-rich leaves containing higher lipid and protein content will more likely be consumed by herbivores before it enters the detrital pool (Cebrián, 1999) or will be rapidly used during decomposition (Canuel and Martens, 1996; Mateo et al., 2006). The holocellulose polysaccharides and non-lignin phenolic compounds, on the other hand, may persist and contribute to C stocks as they are difficult to remineralize in coastal environments (Godshalk and Wetzel, 1978; Enríquez et al., 1993). Non-photosynthetic tissues, which are relatively deplete in labile OM, lipids and proteins, contained more aromatic compounds than leaves and thus have potential to contribute refractory C in blue carbon systems, e.g., a large component of *Posidonia* mattes (Kuo and Cambridge, 1978; Romero et al., 1992; Mateo et al., 1997). It is possible in meadows with high sedimentation rates and rapid burial that the amount of above-ground detritus would be more slowly remineralized under anoxic conditions and thus would enhance blue carbon stocks (Kuo and Cambridge, 1978).

As hypothesized, below-ground biomass is likely to be the highest and most consistent contributor of refractory C in seagrass meadows for several reasons. First, root tissues were found to contain the highest amount of refractory macromolecules in the current study. In some cases, more than 50% of root biomass can be stored in sediments >1 m deep (Serrano et al., 2012) and therefore have potential for significant contributions to blue carbon stocks in seagrass habitats. The storage compounds within the rhizome tissues can be a significant source of labile C to blue C sediments. While it would be expected that this labile C would be easily remineralized, recently discovered pathways of labile OM stabilization through binding to minerals (i.e., adsorption) suggest that some labile C could escape remineralization and contribute to sediment C stocks (Lalonde et al., 2012; Johnson et al., 2015). Still, rhizomes do represent a significant pool of lignocellulose (TI_3_ + TI_4_ = 50–60% DW or lignin/phenolics = 23–30% DW), particularly for species with high total OM content. This pool of refractory carbon would typically be slowly, anaerobically decomposed until the substrate is no longer able to be utilized by microbes through enzymatic or physiochemical limitations (Pellikaan, 1982; Burdige, 2007; Conant et al., 2011). In addition to the living C stocks rhizome and roots tissues being typically higher per area than above-ground tissues (Fourqurean et al., 2012), these below-ground tissues are subjected to less grazing and exportation and are protected from physical removal or disturbances, which would promote further C accumulation. Lastly, it has been shown that below-ground tissues had a high percent (~15%) of biomass pyrolysized >600°C not present in above-ground tissues (Trevathan-Tackett et al., 2015). This fraction of thermally stable biomass was hypothesized to be attributed to sulphation of polysaccharides and a mechanism for enhanced C contribution and sequestration (Trevathan-Tackett et al., 2015).

Preliminary data from another global survey, indicated that temperate seagrasses have more organic C in their living biomass on an areal basis compared to tropical seagrasses (~8.6x; Fourqurean et al., 2012). While we have shown that much of this variation from autochthonous seagrass C could likely be due to greater carbohydrate reserves (labile C) in temperate seagrasses, we would expect much of the labile C would be quickly remineralized by microbes *in situ* (Harrison, 1989; Vichkovitten and Holmer, 2004). Additionally, preliminary data from Fourqurean et al. (2012) showed that there is 5.1-times more sediment organic C per area in temperate than tropical habitats, but deeper sampling by latitude and species is needed to make inferences concerning climatic influences on seagrass C and organic C stocks (i.e., sedimentation, particle trapping). In tropical habitats, grazing may be an important factor that affects OM content and quality by increasing relative refractory OM content (and sequestration potential) via production of cell wall components or aromatic anti-herbivory compounds (Vergés et al., 2008; Steele and Valentine, 2012).

Overall, there was very little variation in the quality of OM content across seagrass taxa within a climatic zone or tissue type. In some cases, i.e., sheaths and stems, morphologically larger taxa, like *Posidonia* and *Enhalus*, have potential to contribute more refractory organics to blue carbon habitats in addition to providing other functions that can enhance C-sequestration like sediment stabilization and particle capture (Duarte and Chiscano, 1999; Orth et al., 2006). However, this is likely to be highly dependent upon the environment, particularly hydrodynamics and allochthonous inputs (Lavery et al., 2013), which could ultimately affect sedimentation rates and vertical growth of the seagrass and thus the amount of organic carbon available for sequestration (Macreadie et al., 2014).

**Figure 12:**
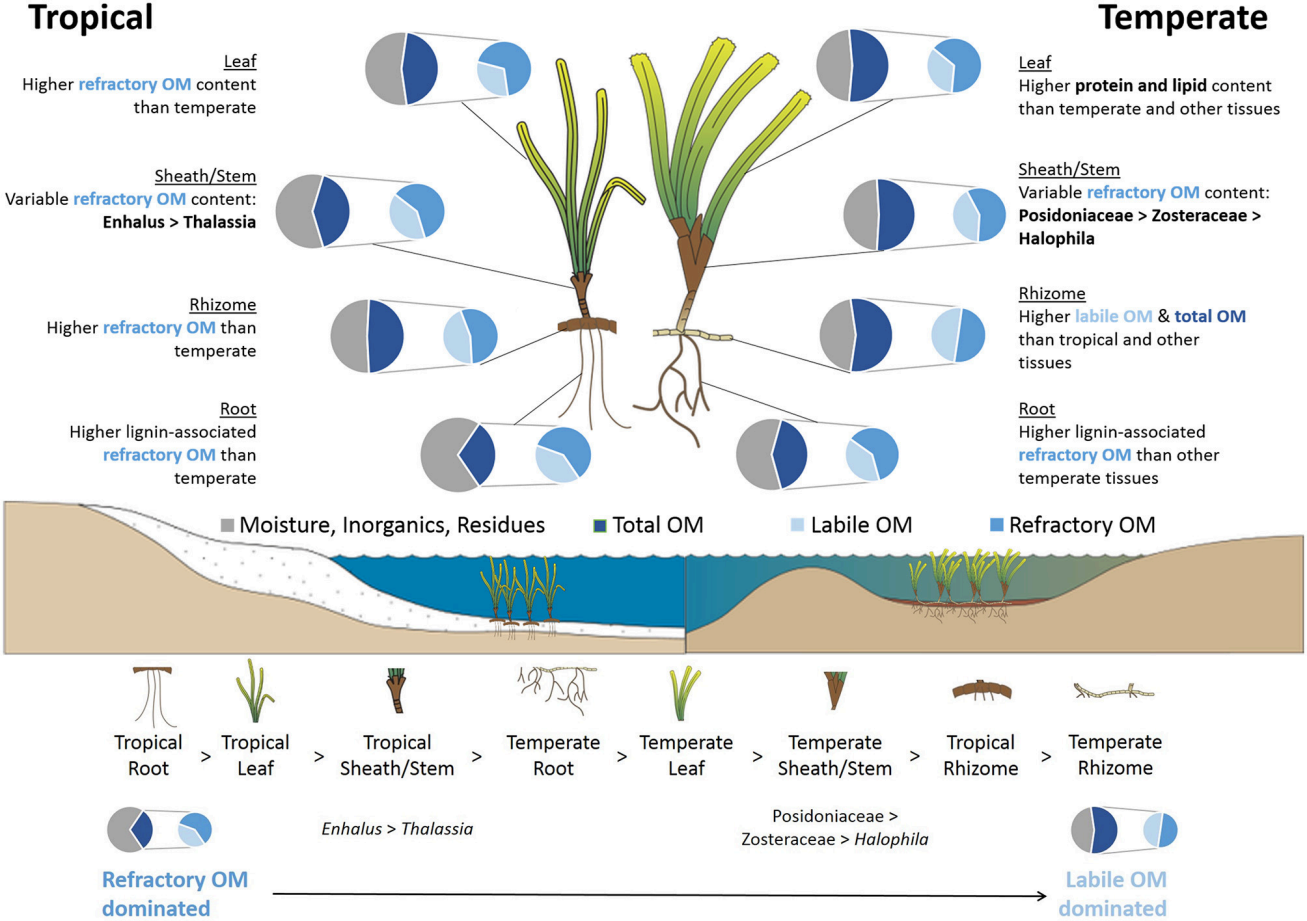
Conceptual model describing the organic matter (OM) quality of seagrasses in different climatic regions, tissue types and taxa based on the thermogravimetric analyses. The model also includes a ranking of seagrass tissues based on refractory OM-dominated to labile OM-dominated characteristics. Total OM is represented by the proportion of total mass that was pyrolysized from 180 to 600°C, while moisture, inorganics and residues are the proportions lost below 180°C and that remained after pyrolysis to 600°C, respectively. Labile OM is the proportion of total OM primarily as soluble carbohydrates and hemicellulose (TI_1_ + TI_2_; 180–300°C), and refractory OM is the proportion of total OM as cellulose and lignin (including possible char residues from pyrolysis, TI_3_ + TI_4_; 300–600°C). Seagrass and landscape mages are courtesy of the Integration and Application Network, University of Maryland Center for Environmental Science (ian.umces.edu/symbols/).

We have provided a conceptual model detailing the variation in seagrass carbon/OM quality (Figure 12). For the sake of simplicity, we focused on using the TGA results to rank the C quality, while understanding that many factors (e.g., microbial remineralization, production and biomass, sediment type and burial rates, etc.) will strongly influence the ultimate fate of seagrass-C sequestration. In summary, we highlighted the importance of considering the location of a seagrass (temperate or tropical) as a driver of C quality, in addition to demonstrating the potential contributions that sheaths and stems can make to C stocks. Future studies further investigating within-climate and within-species variation of C quality will provide valuable insight on the seagrass C contributions to blue carbon ecosystems at a local scale. We encourage the data produced herein to be incorporated in future modeling of C-sequestration processes, and to be used for considerations of blue carbon ecosystem management.

## Author contributions

ST, PM, and PR conceived the ideas and designed methodology; ST, JH, JS, and JB collected and analyzed the data; ST led the writing of the manuscript. All authors contributed critically to the drafts and gave final approval for publication.

### Conflict of interest statement

The authors declare that the research was conducted in the absence of any commercial or financial relationships that could be construed as a potential conflict of interest.
