# Supplementary material for: A Global Assessment of the Chemical Recalcitrance of Seagrass Tissues: Implications for Long-Term Carbon Sequestration
Source: Front Plant Sci. 2017 Jun 13;8:925. doi: 10.3389/fpls.2017.00925 (PMC5468386; doi:10.3389/fpls.2017.00925)
Supplement: Supplementary file 1 [file DataSheet1.DOCX]

Table S1: Review of literature that reports refractory organic matter as a structural carbohydrates and fiber content for above-ground and below-ground seagrass tissues. All data are reported as % dry weight. NDF = neutral detergent fiber (hemicellulose + cellulose + lignin), ADF = acid detergent fiber (cellulose + lignin).

|  |  |  |  |  |  |  |  |  |  |  |  |  |  |
| --- | --- | --- | --- | --- | --- | --- | --- | --- | --- | --- | --- | --- | --- |
|  |  |  |  | Structural Carbohydrates | | |  | Fiber | |  |  |  |  |
| Tissue | Temperate/Tropical | Family | Species | Cellulose | Hemi-cellulose | Lignin |  | NDF | ADF | Reference |  |  |  |
| Leaf | Temperate | Cymodoceaceae | *Amphibolis antarctica* |  |  |  |  | 43.50 |  | 1 |  |  |  |
|  | Temperate | Cymodoceaceae | *Amphibolis griffithii* |  |  |  |  | 47.80 |  | 1 |  |  |  |
|  | Temperate | Cymodoceaceae | *Cymodocea nodosa* |  |  |  |  | 51.00 |  | 2^e^ |  |  |  |
|  | Temperate | Cymodoceaceae | *Cymodocea nodosa* |  |  |  |  | 50.00 |  | 2^e^ |  |  |  |
|  | Temperate | Cymodoceaceae | *Cymodocea nodosa* |  |  |  |  | 46.00 |  | 2^e^ |  |  |  |
|  | Temperate | Cymodoceaceae | *Cymodocea nodosa* |  |  |  |  | 51.00 |  | 2^e^ |  |  |  |
|  | Temperate | Cymodoceaceae | *Syringodium isoetifolium* |  |  |  |  | 38.10 |  | 1 |  |  |  |
|  | Temperate | Cymodoceaceae | *Thalassodendron pachyrhizum* |  |  |  |  | 49.40 |  | 1 |  |  |  |
|  | Temperate | Hydrocharitaceae | *Halophila ovalis* |  |  |  |  | 43.90 |  | 1 |  |  |  |
|  | Temperate | Posidoniaceae | *Posidonia australis* | 20.20 | 11.70 | 14.90^a^ |  | 46.80 | 35.10 | 3 |  |  |  |
|  | Temperate | Posidoniaceae | *Posidonia australis* |  |  |  |  | 57.20 |  | 1 |  |  |  |
|  | Temperate | Posidoniaceae | *Posidonia coriacea* |  |  |  |  | 58.40 |  | 1 |  |  |  |
|  | Temperate | Posidoniaceae | *Posidonia oceanica* | 31.40 | 25.70 | 24.70 |  |  |  | 4 |  |  |  |
|  | Temperate | Posidoniaceae | *Posidonia sinuosa* |  |  |  |  | 59.50 |  | 1 |  |  |  |
|  | Temperate | Zosteraceae | *Zostera marina* | 19.93 |  |  |  |  |  | 5^e^ |  |  |  |
|  | Temperate | Zosteraceae | *Zostera marina* |  |  |  |  | 52.60 |  | 6 |  |  |  |
|  | Temperate | Zosteraceae | *Zostera marina* | 12.20 | 13.80 | 4.30^a^ |  |  |  | 7 |  |  |  |
|  | Temperate | Zosteraceae | *Zostera muelleri* |  |  |  |  | 44.00 |  | 8^e^ |  |  |  |
|  | Temperate | Zosteraceae | *Zostera nigricaulis* |  |  |  |  | 55.20 |  | 1 |  |  |  |
|  | Temperate | Zosteraceae | *Zostera tasmanica* |  |  | 4.90 |  |  |  | 9 |  |  |  |
|  |  |  |  |  |  |  |  |  |  |  |  |  |  |
| *Temperate Avg ± 1 S.E.M* |  |  |  | *20.93 ± 3.95* | *17.07 ± 4.36* | *12.20 ± 4.82* |  | *49.65 ± 1.49* | *35.10 ± 0* |  |  |  |  |
|  |  |  |  |  |  |  |  |  |  |  |  |  |  |
|  | Tropical | Cymodoceaceae | *Cymodocea rotundata* | 13.90^b^ | 17.80^c^ | 19.00^a^ |  | 50.70 | 32.90 | 10 |  |  |  |
|  | Tropical | Cymodoceaceae | *Cymodocea rotundata* |  |  |  |  | 25.20 |  | 11^f^ |  |  |  |
|  | Tropical | Cymodoceaceae | *Cymodocea serrulata* | 13.90^b^ | 17.00^c^ | 15.30^a^ |  | 46.20 | 29.20 | 10 |  |  |  |
|  | Tropical | Cymodoceaceae | *Cymodocea serrulata* |  |  |  |  | 23.93 |  | 11^f^ |  |  |  |
|  | Tropical | Cymodoceaceae | *Cymodocea serrulata* |  |  |  |  | 2.07 |  | 12^ef^ |  |  |  |
|  | Tropical | Cymodoceaceae | *Halodule pinifolia* |  |  |  |  | 16.50 |  | 11^f^ |  |  |  |
|  | Tropical | Cymodoceaceae | *Halodule pinifolia* |  |  |  |  | 4.17 |  | 12^ef^ |  |  |  |
|  | Tropical | Cymodoceaceae | *Halodule uninervis* | 13.70^b^ | 15.60^c^ | 18.90^a^ |  | 48.20 | 32.60 | 10 |  |  |  |
|  | Tropical | Cymodoceaceae | *Halodule uninervis* |  |  | 20.16 |  | 52.50 |  | 13 |  |  |  |
|  | Tropical | Cymodoceaceae | *Halodule wrightii* | 16.15^b^ | 9.50^c^ | 1.85^ad^ |  | 27.50d | 18.00^d^ | 14 |  |  |  |
|  | Tropical | Cymodoceaceae | *Syringodium filiforme* | 13.95^b^ | 8.00^c^ | 2.05^ad^ |  | 24.00d | 16.00^d^ | 14 |  |  |  |
|  | Tropical | Cymodoceaceae | *Syringodium isoetifolium* | 16.50^b^ | 10.30^c^ | 10.20^a^ |  | 37.00 | 26.70 | 10 |  |  |  |
|  | Tropical | Cymodoceaceae | *Syringodium isoetifolium* |  |  |  |  | 21.40 |  | 11^f^ |  |  |  |
|  | Tropical | Cymodoceaceae | *Syringodium isoetifolium* |  |  |  |  | 4.55 |  | 12^ef^ |  |  |  |
|  | Tropical | Hydrocharitaceae | *Enhalus acoroides* |  |  |  |  | 20.30 |  | 11f |  |  |  |
|  | Tropical | Hydrocharitaceae | *Halophila engelmanni* | 12.73 |  | 1.00 |  |  |  | 15e |  |  |  |
|  | Tropical | Hydrocharitaceae | *Halophila minor* | 9.20^b^ | 11.20^c^ | 11.50^a^ |  | 31.90 | 20.70 | 10 |  |  |  |
|  | Tropical | Hydrocharitaceae | *Halophila ovalis* | 11.10^b^ | 10.40^c^ | 10.80^a^ |  | 32.30 | 21.90 | 10 |  |  |  |
|  | Tropical | Hydrocharitaceae | *Halophila ovalis* |  |  | 16.53 |  | 51.14 |  | 13 |  |  |  |
|  | Tropical | Hydrocharitaceae | *Halophila ovalis* |  |  |  |  | 4.42 |  | 12^ef^ |  |  |  |
|  | Tropical | Hydrocharitaceae | *Halophila spinulosa* | 15.80^b^ | 8.10^c^ | 11.20^a^ |  | 35.10 | 27.00 | 10 |  |  |  |
|  | Tropical | Hydrocharitaceae | *Halophila spinulosa* |  |  | 13.99 |  | 52.81 |  | 13 |  |  |  |
|  | Tropical | Hydrocharitaceae | *Thalassia hemprichii* |  |  |  |  | 16.76 |  | 11^f^ |  |  |  |
|  | Tropical | Hydrocharitaceae | *Thalassia testudinum* | 26.53^b^ | 5.22^c^ | 5.92^a^ |  | 37.67 | 32.46 | 16 |  |  |  |
|  | Tropical | Hydrocharitaceae | *Thalassia testudinum* | 24.50^b^ | 8.10^c^ | 2.30^a^ |  | 34.90 | 26.80 | 17 |  |  |  |
|  | Tropical | Hydrocharitaceae | *Thalassia testudinum* | 18.15^b^ | 10.00^c^ | 1.85^ad^ |  | 30.00^d^ | 20.00^d^ | 14 |  |  |  |
|  | Tropical | Hydrocharitaceae | *Thalassia testudinum* | 17.50 | 19.10 | 9.10^a^ |  |  |  | 18 |  |  |  |
|  | Tropical | Zosteraceae | *Zostera muelleri* | 12.60^b^ | 14.30^c^ | 15.40^a^ |  | 42.30 | 28.00 | 10 |  |  |  |
|  | Tropical | Zosteraceae | *Zostera muelleri* |  |  | 18.37 |  | 48.43 |  | 13 |  |  |  |
|  |  |  |  |  |  |  |  |  |  |  |  |  |  |
| *Tropical Avg ± 1 S.E.M* |  |  |  | *15.75 ± 1.20* | *11.76 ± 1.14* | *10.81 ± 1.52* |  | *30.44 ± 3.05* | *25.56 ± 1.58* |  |  |  |  |
|  |  |  |  |  |  |  |  |  |  |  |  |  |  |
| *Total Leaf Avg ± 1 S.E.M* |  |  |  | *16.84 ± 1.29* | *12.70 ± 1.24* | *11.05 ± 1.46* |  | *37.59 ± 2.44* | *26.24 ± 1.62* |  |  |  |  |
|  |  |  |  |  |  |  |  |  |  |  |  |  |  |
|  |  |  |  |  |  |  |  |  |  |  |  |  |  |
| Leaf + Stem | Tropical | Cymodoceaceae | *Cymodocea serrulata* | 27.89^b^ | 21.60^c^ | 6.99^a^ |  | 56.48 | 34.88 | 19 |  |  |  |
|  | Tropical | Cymodoceaceae | *Syringodium isoetifolium* |  |  |  |  | 40.07 | 42.19 | 19 |  |  |  |
|  | Tropical | Hydrocharitaceae | *Halophila ovalis* | 20.42^b^ | 19.04^c^ | 4.13^a^ |  | 43.59 | 24.55 | 19 |  |  |  |
|  | Tropical | Hydrocharitaceae | *Halophila spinulosa* | 28.70^b^ | 13.89^c^ | 4.48^a^ |  | 47.07 | 33.18 | 19 |  |  |  |
|  |  |  |  |  |  |  |  |  |  |  |  |  |  |
| *Total Leaf + Stem Avg ± 1 S.E.M* |  |  |  | *25.67 ± 2.64* | *18.18 ± 2.27* | *5.20 ± 0.90* |  | *46.80 ± 3.53* | *33.70 ± 3.62* |  |  |  |  |
|  |  |  |  |  |  |  |  |  |  |  |  |  |  |
| Non-photosynthetic AG | Temperate | Posidoniaceae | *Posidonia oceanica* | 38.00 | 21.00 | 27.00 |  |  |  | 20 |  |  |  |
|  | Temperate | Zosteraceae | *Zostera tasmanica* |  |  | 2.10 |  |  |  | 9 |  |  |  |
|  | Temperate | Zosteraceae | *Zostera tasmanica* |  |  | 5.40 |  |  |  | 9 |  |  |  |
|  | Tropical | Hydrocharitaceae | *Halophila engelmanni* | 23.63 |  | 0.70 |  |  |  | 15^e^ |  |  |  |
|  |  |  |  |  |  |  |  |  |  |  |  |  |  |
| *Total Non-photosynthetic Avg ± 1 S.E.M* |  |  |  | *30.82 ± 7.18* | *21.00 ± 0* | *8.80 ± 6.15* |  | *N/A* | *N/A* |  |  |  |  |
| *Total AG Avg ± 1 S.E.M* |  |  |  | *19.12 ± 1.49* | *13.87 ± 1.17* | *10.17 ± 1.37* |  | *38.37 ± 2.28* | *27.90 ± 1.62* |  |  |  |  |
|  |  |  |  |  |  |  |  |  |  |  |  |  |  |
| Rhizome | Temperate | Zosteraceae | *Zostera marina* | 18.30 | 28.90 | 3.50^a^ |  |  |  | 7 |  |  |  |
|  | Temperate | Zosteraceae | *Zostera tasmanica* |  |  | 5.40 |  |  |  | 9 |  |  |  |
|  |  |  |  |  |  |  |  |  |  |  |  |  |  |
| *Temperate Avg ± 1 S.E.M* |  |  |  | *18.30 ± 0* | *28.90 ± 0* | *4.45 ± 0.95* |  | *N/A* | *N/A* |  |  |  |  |
|  |  |  |  |  | | |  |  | |  |  |  |  |
|  | Tropical | Cymodoceaceae | *Cymodocea serrulata* |  |  |  |  | 3.22 |  | 12^ef^ |  |  |  |
|  | Tropical | Cymodoceaceae | *Halodule pinifolia* |  |  |  |  | 3.92 |  | 12^ef^ |  |  |  |
|  | Tropical | Cymodoceaceae | *Halodule wrightii* | 12.70^b^ | 11.00^c^ | 1.80^ad^ |  | 25.50^d^ | 14.50^d^ | 14 |  |  |  |
|  | Tropical | Cymodoceaceae | *Syringodium filiforme* | 17.85^b^ | 3.50^c^ | 1.65^ad^ |  | 23.00^d^ | 19.50^d^ | 14 |  |  |  |
|  | Tropical | Cymodoceaceae | *Syringodium isoetifolium* |  |  |  |  | 3.93 |  | 12^ef^ |  |  |  |
|  | Tropical | Hydrocharitaceae | *Halophila engelmanni* | 22.67 |  | 0.47 |  |  |  | 15^e^ |  |  |  |
|  | Tropical | Hydrocharitaceae | *Halophila ovalis* |  |  |  |  | 4.97 |  | 12^ef^ |  |  |  |
|  | Tropical | Hydrocharitaceae | *Thalassia testudinum* | 19.30^b^ | 0.00^c^ | 3.50^a^ |  | 22.80 | 22.80 | 17 |  |  |  |
|  | Tropical | Hydrocharitaceae | *Thalassia testudinum* | 13.35^b^ | 6.00^c^ | 1.15^ad^ |  | 20.50^d^ | 14.50^d^ | 14 |  |  |  |
|  | Tropical | Hydrocharitaceae | *Thalassia testudinum* | 20.20 | 21.10 | 6.80^a^ |  |  |  | 7 |  |  |  |
|  |  |  |  |  |  |  |  |  |  |  |  |  |  |
| *Tropical Avg ± 1 S.E.M* |  |  |  | *17.68 ± 1.61* | *8.32 ± 3.66* | *2.56 ± 0.94* |  | *13.48 ± 3.61* | *17.83 ± 2.03* |  |  |  |  |
|  |  |  |  |  |  |  |  |  |  |  |  |  |  |
| *Total Rhizome Avg ± 1 S.E.M* |  |  |  | *17.77 ± 1.36* | *11.75 ± 4.55* | *3.03 ± 0.78* |  | *13.48 ± 3.61* | *17.83 ± 2.03* |  |  |  |  |
|  |  |  |  |  |  |  |  |  |  |  |  |  |  |
|  |  |  |  |  |  |  |  |  |  |  |  |  |  |
| Root | Temperate | Zosteraceae | *Zostera marina* | 21.30 | 40.90 | 5.70^a^ |  |  |  | 7 |  |  |  |
|  | Temperate | Zosteraceae | *Zostera tasmanica* |  |  | 6.50 |  |  |  | 9 |  |  |  |
|  |  |  |  |  |  |  |  |  |  |  |  |  |  |
| *Temperate Avg ± 1 S.E.M* |  |  |  | *21.30 ± 0* | *40.90 ± 0* | *6.10 ± 0.40* |  | *N/A* | *N/A* |  |  |  |  |
|  |  |  |  |  |  |  |  |  |  |  |  |  |  |
|  | Tropical | Cymodoceaceae | *Cymodocea serrulata* |  |  |  |  | 3.28 |  | 12^ef^ |  |  |  |
|  | Tropical | Cymodoceaceae | *Halodule pinifolia* |  |  |  |  | 2.58 |  | 12^ef^ |  |  |  |
|  | Tropical | Cymodoceaceae | *Syringodium isoetifolium* |  |  |  |  | 2.97 |  | 12^ef^ |  |  |  |
|  | Tropical | Hydrocharitaceae | *Halophila engelmanni* | 13.23 |  | 2.07 |  |  |  | 15^e^ |  |  |  |
|  | Tropical | Hydrocharitaceae | *Halophila ovalis* |  |  |  |  | 3.52 |  | 12^ef^ |  |  |  |
|  | Tropical | Hydrocharitaceae | *Thalassia testudinum* | 23.50 | 28.70 | 5.70^a^ |  |  |  | 7 |  |  |  |
|  |  |  |  |  |  |  |  |  |  |  |  |  |  |
| *Tropical Avg ± 1 S.E.M* |  |  |  | *18.37 ± 5.13* | *28.70 ± 0* | *3.88 ± 1.82* |  | *3.09 ± 0.20* | *N/A* |  |  |  |  |
|  |  |  |  |  |  |  |  |  |  |  |  |  |  |
| *Total Root Avg ± 1 S.E.M* |  |  |  | *19.34 ± 3.12* | *34.80 ± 6.10* | *4.99 ± 0.99* |  | *3.09 ± 0.20* | *N/A* |  |  |  |  |
|  |  |  |  |  |  |  |  |  |  |  |  |  |  |
|  |  |  |  |  |  |  |  |  |  |  |  |  |  |
| Rhizome + Root | Temperate | Zosteraceae | *Zostera marina* | 16.67 |  |  |  |  |  | 5^e^ |  |  |  |
|  | Tropical | Cymodoceaceae | *Cymodocea rotundata* | 12.60^b^ | 9.70^c^ | 19.90^a^ |  | 42.20 | 32.50 | 10 |  |  |  |
|  | Tropical | Cymodoceaceae | *Cymodocea serrulata* | 25.70^b^ | 4.68^c^ | 7.46^a^ |  | 37.84 | 33.16 | 19 |  |  |  |
|  | Tropical | Cymodoceaceae | *Cymodocea serrulata* | 14.40^b^ | 9.30^c^ | 15.80^a^ |  | 39.50 | 30.20 | 10 |  |  |  |
|  | Tropical | Cymodoceaceae | *Halodule uninervis* | 11.60^b^ | 8.30^c^ | 8.40^a^ |  | 28.30 | 20.00 | 10 |  |  |  |
|  | Tropical | Cymodoceaceae | *Halodule uninervis* |  |  | 18.85 |  | 51.41 |  | 13 |  |  |  |
|  | Tropical | Cymodoceaceae | *Syringodium isoetifolium* |  |  | 6.48^a^ |  | 31.09 | 33.55 | 19 |  |  |  |
|  | Tropical | Cymodoceaceae | *Syringodium isoetifolium* | 15.20^b^ | 8.00^c^ | 11.10^a^ |  | 34.30 | 26.30 | 10 |  |  |  |
|  | Tropical | Hydrocharitaceae | *Halophila minor* | 11.00^b^ | 4.50^c^ | 8.80^a^ |  | 24.30 | 19.80 | 10 |  |  |  |
|  | Tropical | Hydrocharitaceae | *Halophila ovalis* | 26.25^b^ | 7.43^c^ | 4.50^a^ |  | 38.18 | 30.75 | 19 |  |  |  |
|  | Tropical | Hydrocharitaceae | *Halophila ovalis* | 12.40^b^ | 5.10^c^ | 9.00^a^ |  | 26.50 | 21.40 | 10 |  |  |  |
|  | Tropical | Hydrocharitaceae | *Halophila ovalis* |  |  | 15.08 |  | 46.53 |  | 13 |  |  |  |
|  | Tropical | Hydrocharitaceae | *Halophila spinulosa* | 30.79^b^ | 13.41^c^ | 4.86^a^ |  | 49.06 | 35.65 | 19 |  |  |  |
|  | Tropical | Hydrocharitaceae | *Halophila spinulosa* | 17.90^b^ | 9.70^c^ | 8.80^a^ |  | 36.40 | 26.70 | 10 |  |  |  |
|  | Tropical | Hydrocharitaceae | *Halophila spinulosa* |  |  | 13.92 |  | 50.09 |  | 13 |  |  |  |
|  | Tropical | Zosteraceae | *Zostera muelleri* | 11.10^b^ | 8.10^c^ | 15.10^a^ |  | 34.30 | 26.20 | 10 |  |  |  |
|  | Tropical | Zosteraceae | *Zostera muelleri* |  |  | 13.19 |  | 43.03 |  | 13 |  |  |  |
|  |  |  |  |  |  |  |  |  |  |  |  |  |  |
| *Total Rhizome + Root Avg ± 1 S.E.M* |  |  |  | *17.13 ± 2.14* | *8.02 ± 0.79* | *11.33 ± 1.19* |  | *38.31 ± 2.10* | *28.02 ± 1.58* |  |  |  |  |
|  |  |  |  |  |  |  |  |  |  |  |  |  |  |
| *Total BG Avg ± 1 S.E.M* |  |  |  | *17.64 ± 1.21* | *12.02 ± 2.41* | *8.05 ± 1.03* |  | *26.19 ± 3.16* | *25.47 ± 1.70* |  |  |  |  |
|  |  |  |  |  |  |  |  |  |  |  |  |  |  |
|  |  |  |  |  |  |  |  |  |  |  |  |  |  |
| Whole plant | Tropical | Cymodoceaceae | *Cymodocea serrulata* | 26.97^b^ | 14.57^c^ | 7.16^a^ |  | 48.70 | 34.13 | 19 |  |  |  |
|  | Tropical | Cymodoceaceae | *Halodule uninervis* |  |  | 19.17 |  | 51.60 |  | 13 |  |  |  |
|  | Tropical | Cymodoceaceae | *Syringodium isoetifolium* |  |  |  |  | 35.05 | 38.21 | 19 |  |  |  |
|  | Tropical | Hydrocharitaceae | *Halophila decipiens* | 15.50^b^ | 7.10^c^ | 4.90^a^ |  | 27.50 | 20.40 | 10 |  |  |  |
|  | Tropical | Hydrocharitaceae | *Halophila ovalis* | 23.05^b^ | 14.16^c^ | 4.10^a^ |  | 41.31 | 27.15 | 19 |  |  |  |
|  | Tropical | Hydrocharitaceae | *Halophila ovalis* |  |  | 15.73 |  | 48.86 |  | 13 |  |  |  |
|  | Tropical | Hydrocharitaceae | *Halophila spinulosa* | 29.44^b^ | 14.17^c^ | 4.56^a^ |  | 48.17 | 34.00 | 19 |  |  |  |
|  | Tropical | Hydrocharitaceae | *Halophila spinulosa* |  |  | 13.54 |  | 50.83 |  | 13 |  |  |  |
|  | Tropical | Hydrocharitaceae | *Halophila trichosata* | 16.60^b^ | 8.40^c^ | 8.10^a^ |  | 33.10 | 24.70 | 10 |  |  |  |
|  | Tropical | Zosteraceae | *Zostera muelleri* |  |  | 14.32 |  | 44.04 |  | 13 |  |  |  |
|  |  |  |  |  |  |  |  |  |  |  |  |  |  |
| *Total Whole Plant Avg ± 1 S.E.M* |  |  |  | *22.31 ± 2.78* | *11.68 ± 1.62* | *10.18 ± 1.86* |  | *42.92 ± 2.65* | *29.77 ± 2.78* |  |  |  |  |
|  |  |  |  |  |  |  |  |  |  |  |  |  |  |
|  |  |  |  |  |  |  |  |  |  |  |  |  |  |

^a^ Lignin reported as acid detergent lignin (ADL).

^b^ Cellulose calculated from ADF – ADL.

^c^ Hemicellulose calculated from NDF – ADF.

^d^ Reference used values of a range, so the median was reported in table.

^e^ Value represents a mean reported over > 1 season.

^f^ NDF was described as TDF.

Literature Cited for Table S1

1 de Los Santos, C. B. *et al.* Leaf-fracture properties correlated with nutritional traits in nine Australian seagrass species: Implications for susceptibility to herbivory. *Mar. Ecol. Prog. Ser.* **458**, 89-102 (2012).

2 de los Santos, C., B, Brun, F. G., Vergara, J. J. & Pérez-Lloréns, J. L. New aspect in seagrass acclimation: leaf mechanical properties vary spatially and seasonally in the temperate species *Cymodocea nodosa* Ucria (Ascherson). *Mar. Biol.* **160**, 1083-1093 (2013).

3 Torbatinejad, N. M., Annison, G., Rutherfurd-Markwick, K. & Sabine, J. R. Structural constituents of the seagrass *Posidonia australis*. *J. Agric. Food Chem.* **55**, 4021-4026, doi:10.1021/jf063061a (2007).

4 Bettaie, F. *et al.* Nanofibrillar cellulose from *Posidonia oceanica*: Properties and morphological features. *Industrial Crops and Products* (2015).

5 Touchette, B. W. & Burkholder, J. M. Seasonal variations in carbon and nitrogen constituents in eelgrass (*Zostera marina* L.) as influenced by increased temperature and water-column nitrate. *Bot. Mar.* **45**, 23-34 (2002).

6 Godshalk, G. L. & Wetzel, R. G. Decomposition of aquatic angiosperms. III. *Zostera marina* L. and a conceptual model of decomposition. *Aquat. Bot.* **5**, 329-354 (1978).

7 Kenworthy, W. J. & Thayer, G. W. Production and decomposition of the roots and rhizomes of seagrasses, *Zostera marina* and *Thalassia testudinum*, in temperate and subtropical marine ecosystems. *Bull. Mar. Sci.* **35**, 364-379 (1984).

8 Nicastro, A., Onoda, Y. & Bishop, M. J. Direct and indirect effects of tidal elevation on eelgrass decomposition. *Mar. Ecol. Prog. Ser.* **456**, 53-62, doi:10.3354/meps09635 (2012).

9 Webster, J. & Stone, B. A. Isolation, structure and monosaccharide composition of the walls of vegetative parts of *Heterozostera tasmanica* (Martens ex Aschers.) den Hartog. *Aquat. Bot.* **47**, 39-52, doi:10.1016/0304-3770(94)90047-7 (1994).

10 Lawler, I. R., Aragones, L., Berding, N., Marsh, H. & Foley, W. Near-infrared reflectance spectroscopy is a rapid, cost-effective predictor of seagrass nutrients. *J. Chem. Ecol.* **32**, 1353-1365, doi:10.1007/s10886-006-9088-x (2006).

11 Rengasamy, R. R. K., Radjassegarin, A. & Perumal, A. Seagrasses as potential source of medicinal food ingredients: Nutritional analysis and multivariate approach. *Biomedicine & Preventive Nutrition* **3**, 375-380 (2013).

12 Jeevitha, M., Athiperumalsami, T. & Kumar, V. Dietary fibre, mineral, vitamin, amino acid and fatty acid content of seagrasses from Tuticorin Bay, Southeast coast of India. *Phytochemistry* **90**, 135-146 (2013).

13 Sheppard, J. K., Lawler, I. R. & Marsh, H. Seagrass as pasture for seacows: Landscape-level dugong habitat evaluation. *Estuar. Coast. Shelf Sci.* **71**, 117-132 (2007).

14 Siegal-Willott, J. L. *et al.* Proximate nutrient analyses of four species of submerged aquatic vegetation consumed by Florida manatee (*Trichechus manatus latirostris*) compared to romaine lettuce (*Lactuca sativa* var. longifolia). *J. Zoo Wildl. Med.* **41**, 594-602 (2010).

15 Dawes, C. *et al.* Proximate composition, photosynthetic and respiratory responses of the seagrass *Halophila engelmannii* from Florida. *Aquat. Bot.* **27**, 195-201 (1987).

16 Bjorndal, K. A. Nutrition and grazing behavior of the green turtle *Chelonia mydas*. *Mar. Biol.* **56**, 147-154, doi:10.1007/bf00397131 (1980).

17 Moran, K. L. & Bjorndal, K. A. Simulated green turtle grazing affects nutrient composition of the seagrass *Thalassia testudinum*. *Mar. Biol.* **150**, 1083-1092, doi:10.1007/s00227-006-0427-9 (2007).

18 Vicente, N., Moreteau, J. C. & Escoubet, P. Etude de l’evolution d’une population de *Pinna nobilis* L. (Mollusque Eulamelibranche) au large de l’anse de la Palud (Parc National de Port-Cros). *Trav. Sci. Parc Natl. Port-Cros* **6**, 39-67 (1980).

19 Sheppard, J. K., Carter, A. B., McKenzie, L. J., Pitcher, C. R. & Coles, R. G. Spatial patterns of sub-tidal seagrasses and their tissue nutrients in the Torres Strait, northern Australia: Implications for management. *Cont. Shelf Res.* **28**, 2282-2291, doi:10.1016/j.csr.2008.03.033 (2008).

20 Ncibi, M. *et al.* Preparation and characterisation of raw chars and physically activated carbons derived from marine *Posidonia oceanica* (L.) fibres. *J. Hazard. Mater.* **165**, 240-249 (2009).
