# Supplementary material for: A Global Assessment of the Chemical Recalcitrance of Seagrass Tissues: Implications for Long-Term Carbon Sequestration
Source: Front Plant Sci. 2017 Jun 13;8:925. doi: 10.3389/fpls.2017.00925 (PMC5468386; doi:10.3389/fpls.2017.00925)
Supplement: Supplementary file 2 [file DataSheet2.DOCX]

Table S2: Raw spectral intensities of main ^13^C-CPMAS NMR functional groups expressed as % of total spectral intensity for selected samples subsample. The spectral ranges integrated for each functional group were amide/carboxyl/ketone (215–165 ppm), O-aromatic (165–145 ppm), aromatic (145–110 ppm), di-O-alkyl (110–95 ppm), O-alkyl (95–60 ppm), N-alkyl/methoxy (60–45 ppm), and alkyl (45 to 10 ppm).

|  |  |  |  |  |  |  |  |  |  |  |
| --- | --- | --- | --- | --- | --- | --- | --- | --- | --- | --- |
| Family | Species | Country | Tissue | Alkyl | N-Alkyl/ Methoxyl | O-Alkyl | Di-O-Alkyl | Aromatic | O-Aromatic | Amide/Ketone/Carboxyl |
| Cymodoceaceae | *Amphibolis antarctica* | Australia, VIC | Leaf | 7.53 | 4.08 | 51.34 | 9.82 | 11.40 | 4.67 | 11.17 |
|  | *Cymodocea nodosa* | Portugal | Leaf | 12.60 | 5.83 | 47.26 | 9.38 | 9.74 | 3.85 | 11.33 |
|  |  |  | Rhizome | 6.01 | 4.99 | 57.10 | 11.54 | 7.53 | 3.76 | 9.08 |
|  |  |  | Root | 9.74 | 5.66 | 49.73 | 10.62 | 9.43 | 4.90 | 9.93 |
|  | *Halodule uninervis* | Australia, QLD | Rhizome | 3.31 | 2.47 | 57.64 | 13.55 | 9.39 | 4.07 | 9.56 |
|  |  |  | Root | 12.08 | 5.52 | 38.45 | 9.36 | 10.68 | 6.10 | 17.81 |
|  | *Syringodium isoetifolium* | Madagascar | Leaf | 11.11 | 4.75 | 47.38 | 10.27 | 10.89 | 4.40 | 11.18 |
|  |  |  | Rhizome | 9.38 | 3.50 | 50.99 | 11.01 | 8.77 | 3.63 | 12.72 |
|  |  |  | Root | 5.61 | 2.32 | 48.38 | 11.24 | 11.65 | 4.90 | 15.90 |
|  | *Thalassodendron ciliatum* | Madagascar | Leaf | 10.73 | 4.48 | 45.46 | 10.27 | 11.85 | 5.12 | 12.09 |
|  |  |  | Vertical Rhizome | 8.92 | 5.56 | 45.75 | 11.35 | 13.59 | 6.33 | 8.51 |
|  |  |  | Rhizome | 5.08 | 4.89 | 47.34 | 12.01 | 14.91 | 7.44 | 8.32 |
|  |  |  | Root | 5.70 | 5.63 | 48.45 | 11.97 | 14.51 | 6.35 | 7.41 |
|  |  |  |  |  |  |  |  |  |  |  |
|  |  |  |  |  |  |  |  |  |  |  |
| Hydrocharitaceae | *Enhalus acoroides* | Australia, QLD | Leaf | 12.92 | 5.04 | 48.98 | 10.87 | 7.98 | 3.18 | 11.04 |
|  |  |  | Sheath | 1.72 | 1.43 | 67.02 | 14.43 | 5.87 | 2.11 | 7.42 |
|  |  |  | Rhizome | 1.98 | 2.45 | 65.52 | 14.43 | 6.16 | 3.09 | 6.37 |
|  |  |  | Root | 7.32 | 3.86 | 55.15 | 11.88 | 7.69 | 3.49 | 10.61 |
|  | *Halophila ovalis* | Australia, QLD | Leaf | 10.08 | 4.50 | 43.92 | 10.00 | 9.96 | 4.13 | 17.41 |
|  |  |  | Rhizome | 5.34 | 2.93 | 52.86 | 11.90 | 8.86 | 3.53 | 14.58 |
|  | *Thalassia hemprichii* | Madagascar | Leaf | 11.12 | 4.64 | 44.98 | 10.51 | 11.54 | 4.55 | 12.65 |
|  |  |  | Rhizome | 5.27 | 2.71 | 52.72 | 12.62 | 11.15 | 4.87 | 10.65 |
|  |  |  | Root | 7.77 | 4.05 | 41.30 | 9.96 | 13.02 | 5.83 | 18.07 |
|  |  |  |  |  |  |  |  |  |  |  |
| Posidoniaceae | *Posidonia australis* | Australia, NSW | Leaf | 10.47 | 3.85 | 46.51 | 11.38 | 12.32 | 5.40 | 10.07 |
|  |  |  | Sheath | 6.73 | 4.42 | 49.71 | 11.67 | 11.96 | 5.64 | 9.87 |
|  |  |  | Rhizome | 6.86 | 4.47 | 49.41 | 11.62 | 13.17 | 6.65 | 7.82 |
|  |  |  | Root | 10.98 | 5.15 | 41.69 | 11.02 | 15.93 | 7.38 | 7.84 |
|  | *Posidonia oceanica* | France | Leaf | 11.16 | 4.20 | 47.26 | 10.57 | 11.69 | 4.45 | 10.67 |
|  |  |  | Sheath | 4.15 | 4.51 | 47.94 | 10.83 | 18.59 | 6.74 | 7.24 |
|  |  |  | Rhizome | 5.32 | 4.50 | 49.81 | 12.11 | 14.70 | 6.85 | 6.70 |
|  |  |  | Root | 6.33 | 4.72 | 49.55 | 10.83 | 16.28 | 6.12 | 6.16 |
|  |  |  |  |  |  |  |  |  |  |  |
| Ruppiaceae | *Ruppia maritima* | Sweden | Leaf | 10.52 | 5.53 | 46.23 | 9.69 | 12.01 | 4.97 | 11.06 |
|  |  |  | Sheath | 5.05 | 2.97 | 55.30 | 11.96 | 10.17 | 4.74 | 9.80 |
|  |  |  | Rhizome | 6.36 | 3.71 | 56.50 | 11.65 | 8.24 | 4.12 | 9.42 |
|  |  |  | Root | 8.60 | 4.27 | 51.42 | 11.33 | 9.85 | 4.01 | 10.52 |
|  |  |  |  |  |  |  |  |  |  |  |
| Zosteraceae | *Zostera muelleri* | Australia, VIC | Leaf | 17.01 | 7.10 | 41.26 | 8.27 | 11.03 | 3.58 | 11.75 |
|  |  | Australia, QLD | Leaf | 11.39 | 4.94 | 46.27 | 9.75 | 12.16 | 3.70 | 11.79 |
|  |  |  | Rhizome | 5.75 | 3.35 | 57.87 | 12.11 | 8.59 | 2.99 | 9.35 |
|  |  |  | Root | 7.43 | 3.60 | 53.45 | 11.38 | 9.14 | 3.44 | 11.55 |
|  | *Zostera nigricaulis* | Australia, VIC | Leaf | 15.95 | 6.41 | 39.73 | 8.95 | 11.00 | 3.66 | 14.30 |
|  |  |  |  |  |  |  |  |  |  |  |
